# How Racial/Ethnic Diversity in Urban Schools Shapes Intergroup Relations and Well-Being: Unpacking Intersectionality and Multiple Identities Perspectives

**DOI:** 10.3389/fpsyg.2020.503846

**Published:** 2020-11-24

**Authors:** Negin Ghavami, Kara Kogachi, Sandra Graham

**Affiliations:** ^1^Department of Psychology, Loyola Marymount University, Los Angeles, CA, United States; ^2^Department of Education, University of California, Los Angeles, Los Angeles, CA, United States

**Keywords:** intersectionality, multiple identities, race/ethnic diversity, sexual orientation, life outcomes

## Abstract

Today’s urban schools provide a unique intergroup context in which the students vary not only by race/ethnicity but also by the relative representation of their racial/ethnic groups. In two studies, we examined how this diversity aligns with intersectionality and multiple identities perspectives to affect the power and status associated with each group to shape intergroup dynamics. Study 1 focused on the *perception* of intergroup bias to investigate how perceived presence of same-race/ethnicity peers affects middle school students’ intersectional intergroup attitudes based on race/ethnicity, gender, and sexual orientation. Middle school students (*N* = 1,107; *M_age_* = 12.10; *SD* = 0.99) were randomly assigned to view Facebook-like profiles of peers that varied by gender (boy, girl), race/ethnicity (African American, Latinx), and sexual orientation (straight, lesbian, gay) and offered their first impressions as a way to assess various domains of intergroup attitudes. The perceived presence of same-race/ethnicity peers influenced intersectional intergroup attitudes, however, differentially so depending on stereotypes, prejudice, and behavioral tendencies. Study 2 focused on the *experience* of intergroup bias and simultaneously examined race, gender, and weight discrimination and its consequences among middle school students (*N* = 4,172; *M_age_* = 13.5; *SD* = 0.87). Using latent profile analysis, five profiles of youth based on the pattern of perceived discrimination due to gender, race/ethnicity, and weight were identified. Being African American, Latinx, and male with a high body mass index (BMI) and few same-race/ethnicity peers at school predicted membership in a race profile, whereas being White or Asian with high BMI and more same-race/ethnicity peers predicted membership in a weight profile. Perceiving oneself as gender atypical was associated with all discrimination profiles.

## Introduction

By 2045, the United States is projected to become a majority-minority nation (U.S. Census Bureau, 2018). This demographic change, however, has already occurred in United States public schools. According to the U.S. Department of Education (National Center for Education Statistics (NCES), 2019), White students comprise 49% of elementary and secondary public school enrollment and these numbers drop to about 7–15% when we focus on urban schools. Although racial/ethnic minority students comprise the numerical majority in United States public schools today, they are not a monolith and represent different racial/ethnic groups who vary in their representation. For example, consider the two largest public school districts in the United States. Whereas the New York City public school district is comprised of 41% Latinx, 26% African American, and 16% Asian students, the Los Angeles Unified School District is 73% Latinx, 8% African American, and 6% Asian students (National Center for Education Statistics (NCES), 2019). This unique intergroup context raises critical questions about the meaning of social identities and how historical majority/minority status intersects with numerical size (status) of those groups to shape students’ experiences. Of course, students do not solely belong to one social group –other meaningful social identities such as gender and sexual orientation are likely to work with race/ethnicity to influence how students are perceived and responded to, which can carry implications for health, well-being, and academic achievement.

Increasingly, researchers have recognized that to fully understand the issues affecting diverse youth today, inclusive theoretical and methodological approaches are needed (e.g., Mays and Ghavami, 2018; Syed et al., 2018). Although developmental psychologists, especially those studying social identities and intergroup relations, have become increasingly aware of the need to move away from a singular and decontextualized approach to development, our theoretical frameworks, methods, and empirical research have not kept pace. Single identity approaches still dominate intergroup theories and methods, for example, focusing on race/ethnicity *or* gender *or* sexuality. These approaches typically draw from Erikson (1968) and social identity theory (Tajfel and Turner, 1986) paradigms, and generate a theory and measure for a particular identity such as race/ethnicity (Cross, 1991; Phinney, 1992; Egan and Perry, 2001; Umaña-Taylor et al., 2004). Other theories such as developmental intergroup theory (Bigler and Liben, 2007; Liben, 2014) propose general processes concerning the ways in which young people form stereotypes and develop intergroup bias about one social identity at a time. In addition to a singular focus, many developmental theories do not explicitly identify, account for, or operationalize the systems of power, privilege, and disadvantage that give meaning and significance to social identities. Even models of the consequences of structural marginalization such as the Minority Stress Model (Meyer, 2003) or Garcia-Coll’s Integrative Model (Garcia-Coll et al., 1996) that do attend to social positions still fall short on explaining how power and privilege shape identities, which in turn, affect youths’ outcomes. As the United States shifts to becoming a majority-minority nation, developmental theories and methods that focus on how multiple identities work together and are influenced by the historical and numerical statuses attached to those identities are essential.

To date, no consensus exists regarding the best way to conceptualize and measure the role of multiple identities in intergroup relations and life outcomes of youth (e.g., Mays and Ghavami, 2018). Two general perspectives have emerged –intersectionality and multiple identities perspectives. An intersectionality perspective asserts that identities fuse to shape experiences whereas a multiple identities perspective states that identities co-occur to affect experiences. There is empirical support for each. Research on intersectional intergroup perception (e.g., Ghavami and Mistry, 2019), for example, demonstrates that early adolescents’ stereotypes of peers’ income are linked to the unique fusion of peers’ race/ethnicity, gender, and sexual orientation. Studies on marginalization based on multiple social identities (e.g., Garnett et al., 2014) show that identities about, for example, race/ethnicity, gender, and SES uniquely configure to shape experiences of discrimination among adolescents. An important yet under-explored area of research is how power dynamics that result from both the historical and numerical statuses of social identities affect intergroup experiences and outcomes.

In the work reported here, we contribute to the discussion of the role of power dynamics in multiple identities and intersectionality research on intergroup relations by focusing on the racial/ethnic context of urban schools. Specifically, we focus on one aspect of the racial/ethnic context –the presence of same-race/ethnicity peers –to capture the numerical status that is linked to perception and experiences of power, privilege, and disadvantage. Centering on the perceptions and experiences of racially/ethnically diverse adolescents attending urban middle schools, we conducted two studies to examine how the number of same-race/ethnicity peers influenced the ways social identities work together to shape intergroup attitudes.

## Intergroup Dynamics and Life Outcomes Among Early Adolescents: Multiple Identities and Intersectionality in Context

Middle school in the United States coincides with early adolescence and marks a critical developmental period for studying intergroup processes. As children enter adolescence, they exhibit greater awareness of social group membership (Ruble et al., 2004), for example of race/ethnicity, gender identity or sexual orientation, and intergroup relations (e.g., Brown, 2017; Ghavami and Peplau, 2018; Graham, 2018). In addition, greater cognitive and social skills bring about abstract ways of thinking about the self as part of the group (Ruble et al., 2004) and allow for a deeper understanding of “us” vs. “them” and the systems of power, privilege, and disadvantage that give meaning to those social groups. Early adolescence and the transition to middle school also correspond with the growing importance of identity negotiation and peer approval among youth who seek to find their niche and fit in (Eccles and Roeser, 2011).

Acknowledging the unique context of early adolescence, a growing number of developmental studies have focused on how identities work in concert to affect youths’ experiences and life outcomes. These studies generally fall under two broad perspectives: multiple identities (e.g., Ashmore et al., 2004) and intersectionality (e.g., Crenshaw, 1995). While both perspectives acknowledge that individuals are members of multiple social groups, these perspectives diverge on *how* identities work together to shape perception, experiences, and outcomes. A multiple identities approach focuses on how identities are organized and ranked within an individual and assumes that individuals have unique identity configurations or hierarchies in which certain identities are more salient or central than others (e.g., Jaret and Reitzes, 1999; Ashmore et al., 2004; Kiang et al., 2008). In support of this view, a number of studies with United States children and early adolescents from diverse racial/ethnic backgrounds including White, African American, Latinx, and mixed-race young people show that gender identity was ranked higher in importance than race/ethnic identity (Turner and Brown, 2007; Rogers and Meltzoff, 2017). This same developmental pattern emerged for native Dutch children (Verkuyten and Thijs, 2001) and with Cambodian, Dominican, and Portuguese immigrant children (Akiba et al., 2004).

Intersectionality also recognizes that individuals belong to multiple social categories but asserts that these categories work together uniquely to form social positions and experiences, which in turn, shape outcomes (e.g., Crenshaw, 1991; Shields, 2008). Therefore, intersectionality is focused more on how the understanding and experience of race/ethnicity, for example, is filtered through the lens of gender or sexual orientation rather than which identity is more central or salient. Key to the definitions of intersectionality is the role of broader power systems that give meaning and significance to social identities. Experiences of advantage or disadvantage based on these identities are presumed to be dependent on the context (Collins, 2000). In line with this view, Ghavami and Mistry (2019) showed that early adolescents’ perceptions of social class depended on particular dimensions of social class. To illustrate, perception of social class position (e.g., rich, poor) was primarily determined by race/ethnicity whereas perception of family income was driven by race/ethnicity, gender, and sexual orientation.

In addition to informing our understanding of intergroup perception, researchers have applied multiple identities and intersectionality models to youths’ outcomes. Some scholars have asserted that individuals who possess two minority statuses should be doubly disadvantaged. This notion was put forth by Black feminists (e.g., Beale, 1970; Bond and Perry, 1970) to describe the everyday experiences of African American women who are doubly disadvantaged in the United States because of both racism and sexism. In support of this hypothesis, Grollman’s (2014) analysis of the National Survey of Midlife Development in the United States showed that multiply disadvantaged adults face a “double disadvantage” in health, in part because of their disproportionate exposure to multiple forms of discrimination. Other scholars (Sidanius and Pratto, 1999; Navarrete et al., 2010) have offered a different view of how identities work together to shape outcomes. As a case in point, Navarrete et al. (2010) asserted that identities intersect to produce unique experiences such that sometimes individuals with one minority identity (e.g., minority men) are at a greater disadvantage than those with multiple minority identities (minority women). These assertions have historically been supported by data from criminal justice and school discipline such as expulsion and suspension rates that document the disproportionate representation of racial/ethnic minority men and boys (e.g., Skiba et al., 2016). Although these perspectives have been useful in uncovering complex dynamics of multiple identities in youths’ lives, little work has assessed the role of context in these processes, namely the power dynamics that give meaning and significance to social identities.

## The Current Studies

In the current work, we begin to address major shortcomings of existing research on multiple identities, intersectionality, and intergroup relations. The applications of multiple identities and intersectionality in developmental science –whether perception or outcomes research –have often been “limited in scope and focus on the individual instead of systems” (Santos and VanDaalen, 2016; pp. 668–669). A decontextualized view of social identities provides an incomplete picture of the complex experiences of diverse youth whose lives unfold in social, historical, and political realities. How do we translate the meaning of systems of power, privilege and disadvantage that give meaning to identities and intergroup relations?

In our work, we offer one conceptualization of the role of power in intergroup processes among early adolescents by focusing on the racial/ethnic context of urban schools. Specifically, we examine the numerical representation of different racial/ethnic groups as an index of power and (dis)advantage. Research with adults demonstrates that *numerical* majority and minority status is tied to intergroup attitudes (e.g., Bettencourt and Bartholow, 1998) and group size has been shown to affect youths’ peer victimization and its sequelae (Juvonen et al., 2006). For example, being a numerical minority can put youth at greater risk for marginalization and social isolation (Kogachi and Graham, 2020).

Another major limitation of the extant developmental intergroup research is that it has historically focused narrowly on intergroup dynamics between White-Black youths, though increasingly researchers have examined the experiences of Latinx (e.g., Espinoza et al., 2016) and Asian/Asian American youths (e.g., Chen and Graham, 2015). The shift in United States racial/ethnic demographics raises important questions about the meaning of minority and majority statuses and calls into question the utility of a binary focus on intergroup dynamics. To extend previous work, our research is situated in urban schools where the student body varies not only based on race/ethnicity but also on the relative representation of each racial/ethnic group. This unique intergroup context allows us to assess how numerical status in conjunction with race/ethnicity affects intergroup dynamics –not only among African American and White youths but also among Asian American and Latinx youths.

Finally, because at present no consensus about how best to translate the insights of intersectionality to the conduct of empirical research exists (e.g., Cole, 2009), creating developmentally appropriate and meaningful methods to measure intergroup perceptions and experiences in context is critical. In particular, because most research on perception of youth from diverse backgrounds has taken a singular approach and centered on either race/ethnicity, gender, or sexual orientation, and none (to our knowledge) has assessed the role of racial/ethnic diversity in these intersectional processes, we developed an age-appropriate method to examine intersectional intergroup perceptions.

Integrating research from social (e.g., Bettencourt and Bartholow, 1998) and developmental (Kogachi and Graham, 2020) psychology, we investigate how the presence of same-race/ethnicity peers (i.e., group size) influences intergroup perceptions and experiences. We draw on intersectionality (e.g., Crenshaw, 1995) and multiple identities (e.g., Ashmore et al., 2004) frameworks to assess how identities are configured and intersected and their implications for youths’ outcomes in the context of the presence of same-race/ethnicity peers. Two studies with middle school students are reported, each including multiple racial/ethnic groups recruited from middle schools in urban districts. In Study 1, we focus on *perception* and examine how perceived presence of same-race/ethnicity peers affects early adolescents’ intersectional stereotypes, intergroup emotions, and behavioral tendencies based on race/ethnicity, gender, and sexual orientation of the peers. Study 2 targets the *experience* of bias and investigates how actual presence of same-race/ethnicity peers shapes the configuration of race, gender, and weight discrimination and its consequences for well-being and academic achievement.

## Study 1: How Perceived Presence of Same-Race/Ethnicity Peers Shapes Intersectional Intergroup Attitudes

Numerical status influences intergroup attitudes. Social psychological research with adults shows that majority and minority group members exhibit different types of intergroup attitudes and bias (e.g., Bettencourt and Bartholow, 1998). Majority group members, for example, tend to *emphasize* differences between ingroups and outgroups and this tendency is due to a need for differentiation. By contrast, minority group members tend to *deemphasize* differences between ingroups and outgroups, presumably a reflection of their need to affiliate.

Available studies examining status-based patterns of intergroup attitudes and bias have primarily focused on a single social category and separating numerical and historical statuses has been challenging because they are often confounded. In addition, most studies have focused on race/ethnicity (e.g., race: Whites vs. African Americans). Consequently, it is unclear how numerical status of a racial/ethnic group works together with its historical status to affect intergroup attitudes and bias, and whether those patterns change when other social categories such as sexual orientation are taken into account. For instance, would a lesbian or gay peer of one’s own racial/ethnic group be perceived as an “ingroup” based on shared race/ethnicity or as an “outgroup” based on minority sexual orientation? Importantly, how might perceived presence of same-race/ethnicity peers affect ingroup/outgroup perceptions and implications for intergroup attitudes? The answer to these questions will shed light on whether perceived group size will enhance, attenuate or leave unchanged differences in intergroup attitudes at the intersection of race/ethnicity, gender, and sexual orientation.

We draw on an intersectionality framework to investigate how perceived racial/ethnic group size shapes self-identified straight Latinx and African American urban middle school students’ intersectional intergroup attitudes. We focus on Latinx and African American students because both racial/ethnic groups are historical minority groups in the United States, albeit with distinct social, historical, and political realities. In California, Latinx are in the numerical majority and African Americans in the numerical minority. This numerical majority-minority status is reflected in the student body of the school district from which we recruited our participants.

We use the BIAS MAP (Cuddy et al., 2007) as a method to assess intergroup attitudes about straight and lesbian and gay boys who varied in race/ethnicity. Moreover, we used this framework as a way to focus on how a group’s status in an intergroup context affects how that group is treated and responded to. Urban middle school participants were randomly assigned to view different Facebook-like profiles of fictitious peers that varied systematically based on race/ethnicity, gender (boy, girl), and sexual orientation (straight, lesbian or gay). After viewing each profile, students were asked about their “first impressions” of the featured student to assess gender typicality stereotypes, intergroup status emotions, and behavioral tendencies.

Drawing on prior literature on group size and intergroup bias (Bettencourt and Bartholow, 1998), we generated two main hypotheses. Hypothesis 1 asserts that as perceived size of one’s own racial/ethnic group increases so does the perceived differences between groups. Because research has primarily focused on one social category as it relates to group size in that type of intergroup bias, we were agnostic about how perceived group size would affect the nature of intersectional intergroup attitudes. We also examined whether and how perceived group size shaped intersectional intergroup attitudes differently depending on the intergroup domain. In Hypothesis 2, we assert that different dimensions of intergroup attitudes would be uniquely sensitive to the role of perceived presence of same-race/ethnicity peers. Specifically, Hypothesis 2a states that given that gender typicality stereotypes measure consensually held beliefs about groups, perceived group size may not affect them. By contrast, Hypothesis 2b states that because intergroup emotion and behavioral tendencies implicate the self, perceived presence of same-race/ethnicity peers may exert a greater influence.

### Methods

#### Participants

Early adolescents (*N* = 1,107) who self-identified as straight and either Latino/a^1^ (*n* = 904), or as Black/African American (*n* = 203) enrolled in 6th through 8th grades from four public urban middle schools in the southwestern United States participated. Participants were proficient in English and ranged in age from 10 to 15 years (*M* = 12.35; *SD* = 1.00). To assess gender, participants were asked if they are a “boy,” “girl,” “transgender” or “unsure” of their gender. Based on self-report, 57% identified as a girl, 43% as a boy, and five students indicated that they were unsure of their gender. No participant self-identified as transgender. Most participants (87%) were born in the United States but, on average, 70% reported having at least one parent who was born outside of the United States. The district from which we recruited our sample was comprised of majority Latinx students (Latinx students ∼87% of student body). This racial/ethnic demographic characteristic is reflected in our sample. The schools varied in the actual percent representation of each racial/ethnic group. Latino/a students comprised the largest ethnic group at each of the four schools ranging from 42 to 63% (*M* = 52%). Black/African American students were in the numerical minority in all of the schools ranging from 17 to 35% (*M* = 25%). All four schools qualified for Title I^2^ compensatory education funding.

#### Procedures

All procedures were approved by the Internal Review Board of the school district as well as the University. Active parental consent was obtained and student assent was obtained from those students who received parental consent. The survey was given to participating students during their Science or Health classes and students were debriefed in their respective classrooms. Students did not receive any incentive but were entered into a raffle to win school supplies.

##### Stimulus construction to assess intergroup attitudes

To assess intergroup attitudes, we created Facebook-like profiles. Participants were told that the researchers were interested in assessing their “first impressions” about other students based on their Facebook profiles. In reality, these profiles were fictitious. Using the format of Facebook, each profile contained a headshot photo, name to denote race/ethnicity, and sex. Although not explicitly stated, each profile also communicated the sexual orientation of the featured student. Relying on the format of FB, we indicated who the FB student was interested in dating—interested in dating boys, girls, or both boys and girls. By crossing gender of the FB student (e.g., girl) with gender of the person the FB student is interested in dating (e.g., girls), we created the sexual orientation variable (e.g., lesbian = a female FB student interested in dating girls). Crossing these factors resulted in 24 possible conditions: 2 (gender of FB student) by 3 (sexual orientation) by 4 (race/ethnicity: Asian, African American, Latinx, and White). Additionally, the profiles contained distractor information including a fictitious name of the middle school the student attended and a fictitious city of residence. All photos were pilot tested with a separate group of urban middle school students and standardized based on gender typicality, level of attractiveness, and race/ethnic stereotypicality (for description see Ghavami and Peplau, 2018). To ensure that participants accurately perceived the social category membership of the student in each profile, three manipulation-check questions were asked that corresponded to the race/ethnicity, gender, and sexual orientation of the student in each FB profile.

To minimize participant fatigue, we employed a well-established procedure used by social psychological (e.g., Cuddy et al., 2007) and developmental (e.g., Ghavami and Peplau, 2018) studies where each participant was randomly assigned to review 5 of the 24 possible combinations, and asked to offer their first impressions of each of those 5 profiles. Two constraints were imposed on the composition of the sets of 5 FB-like profiles. First, within each group of 5 profiles, pictures were not repeated and all four races/ethnicities had to be represented with no two successive profiles representing the same racial/ethnic group. Second, all sexual orientations had to be represented within each group of 5 profiles: a straight boy, a straight girl, a lesbian girl, a gay boy and either a bisexual boy or girl. Because so little attention has been given to early adolescents’ attitudes toward bisexual peers at the intersection of race/ethnicity and gender (see Ghavami and Peplau, 2018 for exception), we included FB profiles of bisexual youths and placed those FB profiles last as a first step to explore whether middle school students have distinct and differentiated attitudes toward bisexual peers. In the current analysis, we focus only on the profiles of peers who were straight, gay, and lesbian (total of 16 possible conditions) given the complexity of our research design and the lack of prior research on intergroup attitudes toward bisexual individuals that would help in making predictions. Matching our participants’ race/ethnicity, we limited analyses to targets who were Latinx or African American. After viewing each profile, participants were asked questions about their impressions of the student featured in the FB-profile to assess stereotypes, intergroup emotions, and behavioral tendencies.

#### Measures

To measure perceived *gender typicality*, we followed Ghavami and Peplau (2018) and used two separate items, one to assess perceived masculinity (similarity to boys) and one to assess perceived femininity (similarity to girls). Two questions assessing *intergroup status emotions* were included. One was “If you were to guess, would you want to be like this student?” and the other was “If you were to guess, would you respect this student?” Two questions assessing *positive intergroup behavioral tendencies* were included. One was “If you were to guess, would you sit with this student at lunch or nutrition?” and the other was “If you were to guess, would you invite this student to parties?” Each item was rated on a 5-point scale from 1 (*no way!*) to 5 (*definitely yes!*) and scores were averaged across participants to calculate mean scores for each domain of intergroup attitudes, namely, for similarity to boys, similarity to girls, intergroup status emotions and behavioral tendencies.

##### Perception of percent same-race/ethnicity peers present at school

Participants were asked one question about how many students of their own ethnic group they believed were at their school. This item was rated on a 7-point scale from 1 (*none or hardly any (less than 10%)*) to 7 (*all or almost all (90–100%)*).

### Results

The main goal of this study was to examine whether and how perceived presence of same-race/ethnicity peers shaped intersectional intergroup attitudes among Latinx and African American middle school students. Because of the random assignment of participants to a subset of the FB profile conditions and because intergroup attitudes were measured on a continuous scale, a series of Linear Mixed Models (LMM; West et al., 2006) with random intercepts were conducted. LMM accounts for non-independence among repeated observations that are measured on a continuous scale. Restricted Maximum Likelihood (RML) was used to estimate the models. LMM is a multilevel analog of repeated measures Analysis of Variance (ANOVA) and was conducted in SPSS v. 24 (IBM Corp, 2013) and includes all possible comparisons.

In these LMM models, the fixed effects included the main effects for the FB-profile students’ race/ethnicity and gender as well as the main effect of perceived percent same race/ethnicity peers. Note that for analytic and reporting purposes, FB students’ sexual orientation was created by crossing the gender of the FB student with the gender of the person the FB student is interested in dating (e.g., lesbian = a girl who is interested in dating girls), creating an intersectional variable. As shown in Table 1, because the main purpose of the current study was to examine the role of perceived presence of same-race/ethnicity peers in intersectional intergroup attitudes, the LMM analysis included only those interaction terms that included the FB variables and perceived presence of same-race/ethnicity peers (e.g., FB gender x perceived same-race/ethnicity peers, FB race/ethnicity x sexual orientation x perceived same-race/ethnicity peers). All analyses controlled for participants’ gender. Because little is known about the role of perceived presence of same race/ethnicity peers and intersectional intergroup attitudes among Latinx and African American youth, we first examined these associations separately for each race/ethnic group. However, our analyses yielded comparable results for Latinx and African American participants, both with respect to the mean levels on the variables of interest and the correlations among them. Therefore, we combined the data for Latinx and African American participants and report results for the total sample below. Subsequent pairwise comparisons were conducted with Bonferroni corrections. Because LMM is a multi-level analog of a repeated measures ANOVA, means and standard errors are reported. Table 1 shows the coefficients from final Linear Mixed Models of intergroup attitudes as a function of FB profiles’ race/ethnicity, gender, and sexual orientation.

**TABLE 1:** Coefficients from final linear mixed models of intergroup attitudes as a function of facebook student profiles’ race/ethnicity, gender, and sexual orientation.

	Similarity to girls				Similarity to boys				Status emotions				Positive behavioral tendencies			
				
	Coef	SE	*t*	*p*	Coef	SE	*t*	*p*	Coef	SE	*t*	*p*	Coef	SE	*t*	*p*
**Participant variables**																
Gender (girl omitted)	0.08	0.05	1.66	0.10	−0.01	0.05	−0.18	0.85	−0.13	0.03	−4.694	< 0.001	−0.14	0.05	−2.79	< 0.05
**FBprofiles variables**																
FB Gender (girl omitted)	−0.97	0.21	−4.65	< 0.001	1.1	0.16	7.02	< 0.001	−0.06	0.07	−0.85	0.39	−0.06	0.12	−0.47	0.64
FB Race/ethnicity (Latinx omitted)	−0.212	0.15	−1.45	0.15	−0.20	0.15	−1.37	0.17	0.15	0.06	2.62	< 0.05	−0.02	0.10	−0.16	0.88
FB Sexual orientation	1.36	1.5	9.46	< 0.001	−1.05	0.15	−7.05	< 0.001	0.02	0.06	0.35	0.73	0.22	0.10	2.20	< 0.05
Perceived presence of own ethnicity peers	0.02	0.04	0.63	0.53	−0.01	0.04	−0.35	0.73	−0.01	0.02	−0.56	0.57	0.05	0.03	1.60	0.11
FB Gender X own ethnicity peers	0.09	0.05	1.83	0.07	−0.09	0.04	−2.23	< 0.05	0.03	0.08	1.87	0.06	−0.01	0.03	−0.40	0.69
FB Race/ethnicity x own ethnicity peers	0.02	0.04	0.68	0.50	0.06	0.04	1.65	0.10	−0.05	0.02	−3.15	< 0.05	−0.04	0.03	−1.43	0.15
**FB sexual orientation x own ethnicity peers**																
Gay boys (straight boys omitted)	−0.10	0.05	−1.78	0.08	0.04	0.04	1.00	0.32	−0.05	0.02	−3.08	< 0.05	−0.07	0.03	−2.69	< 0.05
Straight girls (lesbian girls omitted)	−0.12	0.05	−2.43	< 0.05	0.08	0.04	2.12	< 0.05	0.03	0.02	1.96	< 0.05	0.02	0.03	0.72	0.47
**FB Race x sexual orientation x own ethnicity peers**																
Black gay boys	0.002	0.03	0.08	0.94	−0.02	0.03	−0.65	0.52	0.01	0.01	0.64	0.52	0.01	0.23	0.48	0.63
Black straight boys	−0.001	0.03	−0.05	0.96	0.01	0.03	0.43	0.67	0.01	0.01	0.75	0.46	0.04	0.22	1.65	0.10
Black straight girls	−0.01	0.03	−0.42	0.68	0.02	0.03	0.78	0.43	0.01	0.01	0.94	0.35	−0.20	0.24	−0.73	0.47

#### What Is the Role of Perceived Presence of Same-Race/Ethnicity Peers at School on Gender Typicality Stereotypes?

We found no significant main effect of participants’ gender on ratings of gender typicality (all *p*s = n.s.). Our analysis failed to reveal a significant interaction between FB race/ethnicity, sexual orientation (i.e., FB gender X gender of dating partner), and perceived presence of same-race/ethnicity peers on either similarity to boys (*F*(3,2157) = 0.67, *p* = 0.57) or similarity to girls (*F*(3,2163) = 0.09, *p* = 0.96).

**FIGURE 1:**
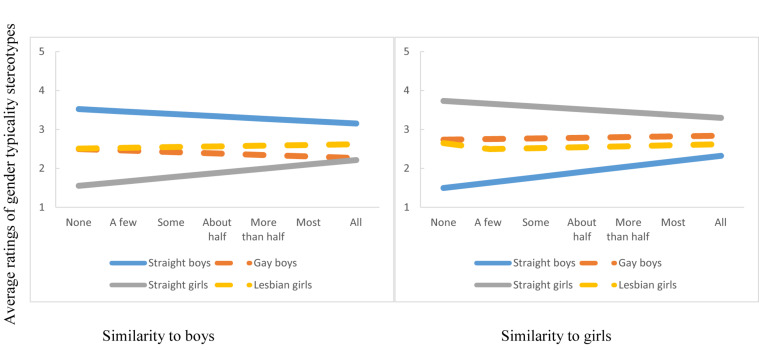
Participants’ ratings of FB profiles on similarity to boys and to girls at the intersection of gender and sexual orientation.

As shown in Figure 1, we found a significant interaction between sexual orientation of FB-profiles and perceived presence of same-race/ethnicity peers on perceived similarity to boys (*F*(2,1811) = 7.37, *p* < 0.05), and on perceived similarity to girls (*F*(2,1794) = 6.04, *p* < 0.05). As perceived presence of same-race/ethnicity peers increased, so did the *differences* in ratings of gender typicality between straight vs. gay and lesbian FB profiles. Specifically, the FB profiles of lesbian and gay students were rated as significantly more gender atypical than the FB profiles of their straight counterparts. Consistent with Hypothesis 2a, the ratings of gender typicality for gay and lesbian FB profiles remained consistent regardless of how many same-ethnicity peers were perceived to be present at school. By contrast, those ratings changed for straight FB profiles. The more same-race/ethnicity peers were perceived to be present, the more gender atypical the straight FB profiles were rated to be. The patterns reported above emerged irrespective of whether the FB profiles were depicting Latinx or African American students. While these findings did not support Hypothesis 1 (stronger differentiation in bias as group size increased), we found support for Hypothesis 2a, specifically, as it related to gender typicality stereotypes of lesbian and gay FB profiles.

#### What Is the Role of Perceived Presence of Same-Race/Ethnicity Peers at School on Intergroup Status Emotions?

We found a significant main effect of participants’ gender on ratings of intergroup status emotions (*F*(1,1074) = 22.04, *p* < 0.001) with girls (*M* = 3.02, *SE* = 0.02) reporting significantly higher ratings of respect and admiration than did boys (*M* = 2.88, *SE* = 0.02). Our analysis failed to reveal a significant interaction between FB race/ethnicity, sexual orientation (i.e., FB gender X gender of dating partner), and perceived presence of same-race/ethnicity peers on intergroup status emotions (*F*(3,2115) = 0.33, *p* = 0.80).

**FIGURE 2:**
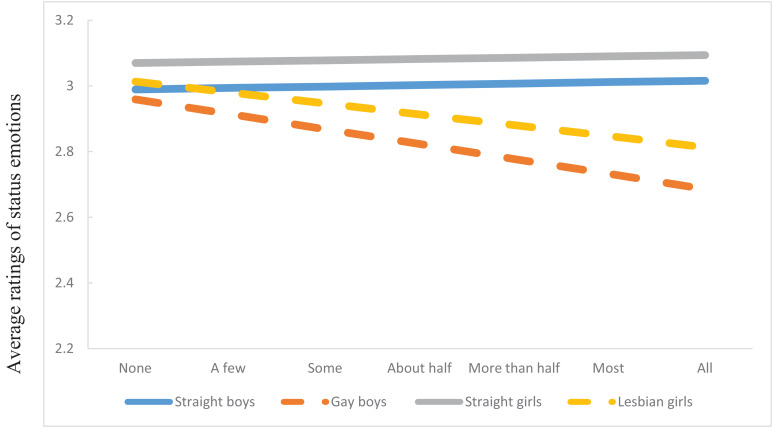
Participants’ ratings of intergroup status emotions at the intersection of gender and sexual orientation.

As shown in Figure 2, analysis revealed a significant interaction between sexual orientation of FB-profiles (i.e., FB gender X gender of dating partner) and perceived presence of same-race/ethnicity peers on intergroup status emotions (*F*(2,1540) = 52.78, *p* < 0.001). In line with Hypothesis1, as perceived presence of same-race/ethnicity peers increased so did the *differences* in ratings of respect and admiration toward straight vs. gay and lesbian FB profiles. The patterns reported above emerged irrespective of whether the FB profiles were depicting Latinx or African American students.

#### What Is the Role of Perceived Presence of Same-Race/Ethnicity Peers at School on Positive Intergroup Behavior?

We found a significant main effect of participants’ gender on ratings of positive intergroup behavior (*F*(1,1081) = 7.81, *p* < 0.001) with girls (*M* = 2.85, *SE* = 0.03) reporting significantly more positive intergroup behavior than boys (*M* = 2.71, *SE* = 0.04). Our analysis failed to reveal a significant interaction between FB race/ethnicity, sexual orientation (i.e., FB gender X gender of dating partner), and perceived presence of same-race/ethnicity peers on intergroup behavior (*F*(3,2087) = 2.07, *p* = 0.10).

**FIGURE 3:**
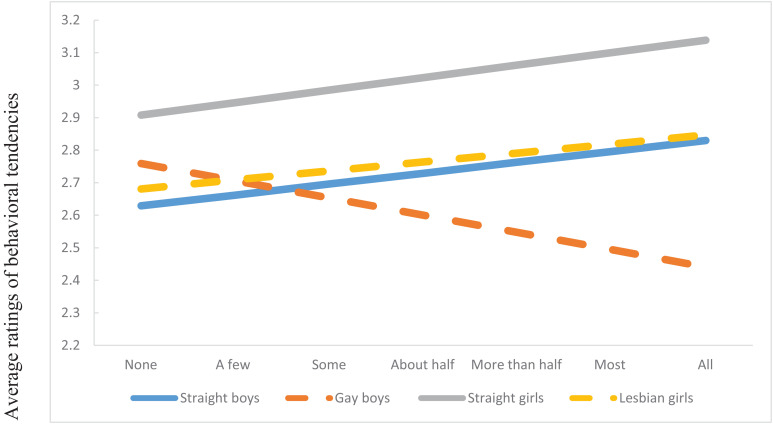
Participants’ ratings of behavioral tendencies at the intersection of gender and sexual orientation.

As shown in Figure 3, analysis revealed a significant interaction between sexual orientation of FB-profiles (i.e., FB gender X gender of dating partner), and perceived presence of same-race/ethnicity peers on positive intergroup behavior (*F*(2,1517) = 23.62, *p* < 0.001). Consistent with Hypothesis 1, as perceived presence of same-race/ethnicity peers increased, so did the *differences* in ratings of positive intergroup behavior among target groups, with gay boy FB profiles being rated especially low on positive intergroup behavior the more same-race/ethnicity peers were perceived to be present. The patterns reported above emerged irrespective of whether the FB profiles were depicting Latinx or African American students.

In summary, we found that perceived presence of same-race/ethnicity peers shaped intersectional intergroup attitudes, however, differentially so depending on the domain of the intergroup attitudes –patterns that were consistent with Hypothesis 2. In particular, consistent with Hypothesis 2a, ratings of gender typicality associated with lesbian and gay FB peers did not significantly change as a function of perceived group size. By contrast, and consistent with Hypothesis 2b, while differences in ratings of both positive emotions and behavior toward various target groups of FB profiles increased as the perceived group size increased, gay boy FB profiles were rated especially low on positive intergroup behavior the more same-race/ethnicity peers were perceived to be present. The “fanning out” effect of intersectional intergroup status emotions and behavior are consistent with our Hypothesis 1.

### Discussion

In this study, we examined self-identified straight African American and Latino/a youths’ intergroup attitudes at the intersection of race/ethnicity (African American and Latinx), gender (boy, girl) and sexual orientation (straight, gay, lesbian). Importantly, we assessed whether perceived presence of same-race/ethnicity peers influenced the nature of intersectional intergroup attitudes. Our results demonstrated that for the three domains of intergroup attitudes –gender typicality stereotypes, status emotions, and behavioral tendencies –gender and sexual orientation (and not race/ethnicity) of the FB profiles drove early adolescents’ perceptions. It is important to note that our findings do not suggest that the race/ethnicity of FB profiles does not matter. Rather, these results suggest that when social identities are considered jointly in the context of perceived presence of same-race/ethnicity peers, race/ethnicity of the FB profiles moves to the background while gender and sexual orientation of those profiles move to the fore to determine intergroup attitudes –insights we would have missed had we only focused on one identity and without attention to context.

Consistent with our Hypothesis 2, the perceived presence of same-race/ethnicity peers influenced the nature of intersectional intergroup attitudes, however, differentially so depending on the domain. For gender typicality stereotypes, in line with Hypothesis 2a, perceived presence of same-race/ethnicity peers did not influence gender typicality ratings of lesbian and gay FB profiles. Irrespective of the perceived group size or whether they were rating a racial/ethnic ingroup or outgroup, African American and Latinx respondents rated lesbian and gay FB profiles as more gender atypical than straight FB profiles. Although no studies have examined the nature of intersectional intergroup stereotypes in the racial/ethnic context, our findings are consistent with developmental studies demonstrating that adolescents perceive gay and lesbian youth as more gender atypical than straight youth, irrespective of those peers’ race/ethnicity (e.g., Ghavami and Peplau, 2018). In addition, our results are in line with adult research that documents a strong consensus about “gender inversion” of gay men and lesbians (Kite and Deaux, 1987) – that gay men are perceived as more feminine and lesbians as more masculine than their heterosexual counterparts. Similar patterns have been documented among early adolescents (e.g., Ghavami and Peplau, 2018). Our study extends these findings by demonstrating that the gender inversion stereotypes are not only present among early adolescents but that they appear to be insensitive to the size of one’s racial/ethnic group.

Interestingly, however, perceived group size did affect the gender typicality of straight FB profiles. When all peers were perceived to be of one’s own racial/ethnic background, the ratings of gender atypicality of straight FB profiles came close to those of gay and lesbian FB profiles. These findings were unexpected and provide insights into the nature of bias at the intersection of gender and sexual orientation in the race/ethnic context. While gay and lesbian peers are “othered” no matter how many racial ingroups are perceived to be present, the imposition of otherness for straight peers emerges when the context is mostly racially/ethnically homogeneous. Gender atypicality is often perceived as a dimension of difference, a difference that is stigmatized (e.g., Kite and Deaux, 1987). Studies have shown that children and youth use terms such as “that’s so gay” or “fag” to bully and victimize gender non-conforming youth –associating gender atypicality with minority sexual orientation, a status that is stigmatized in the United States (e.g., Toomey et al., 2013). With larger group size comes power and need for differentiation, and in our sample, the participants may have been more willing to show bias and distance themselves from other straight peers by rating them as gender atypical.

A different picture emerged for intersectional positive intergroup emotions, and behavioral tendencies. Though gender and sexual orientation of the FB profiles still drove ratings of admiration, respect, and willingness to interact with peers, as perceived group size increased, so did the differences in ratings of target groups. This “fanning out” effect is consistent with our Hypothesis 1 and prior research on majority status, intergroup attitudes, and bias (e.g., Bettencourt and Bartholow, 1998). The specific pattern of fanning out is noteworthy, however. The FB profiles of straight girls were rated as most respected and admired and received the greatest willingness for interaction. These findings are consistent with developmental research (e.g., Ghavami and Peplau, 2018) that show that straight girls are generally perceived positively when rated on such stereotypes as being smart. By contrast, the FB profiles of gay boys received the lowest ratings of positive intergroup emotions and behavior but particularly so for the behavioral measures. Those of straight boy and lesbian girl FB profiles fell in between those two groups. The findings for gay boys are also consistent with prior research –both with adults as well as youth. For example, studies consistently demonstrate (e.g., Kite and Deaux, 1987; Brown, 2017) that men and boys who move away from prescribed gender norms are vulnerable to experiencing social exclusion, bullying, and victimization. Our results add to prior research by uncovering similar patterns of bias, most strongly in the behavioral domain, in the racial/ethnic context, and at the intersection of social identities.

Taken together, our results suggest that perceived numerical status shifts the meaning and significance of social categories at the intersections and changes how identities work together to shape intergroup bias. Our findings provide initial evidence that when historical minority respondents perceive themselves as the numerical majority, they display patterns of bias that are consistent with those found traditionally among majority group members. In addition, the influence of group size in bias may be sensitive to other features of the social context, in this case, the domain of intergroup attitudes (e.g., emotion vs. behavior). In the next study, we further disentangle societal and numerical status using a multiple identities perspective. We examine the experience of discrimination across multiple social identities among societal high status (White and Asian) and low status (African American and Latinx) early adolescents who vary in numerical size at their middle school.

## Study 2: How Presence of Same-Race/Ethnicity Peers Shapes Profiles of Discrimination Experiences and Implications for Well-Being and Academic Achievement

Experiences of discrimination in school are part of the everyday life of many youth and those experiences can take their toll on the mental health, physical health, and academic well-being of youth. Most research has focused on racial discrimination, or unfair treatment attributed to one’s race or ethnicity (e.g., Benner et al., 2018; Seaton et al., 2018). We know that youth of color who experience race-based discrimination often report feeling depressed and anxious and some do more poorly in school (Benner et al., 2018). In the context of discrimination, race/ethnicity can be conceptualized as a social stigma—a social identity that is devalued in the eyes of others (Crocker et al., 1998). There are other devalued social identities possessed by youth, such as gender atypicality, sexual minority status, or obesity, that also elicit discrimination and that have known consequences not unlike those linked to racial discrimination. Being picked on because you are overweight (e.g., Puhl and Latner, 2007; Schvey et al., 2014) or because you deviate from the gender norms in your school (e.g., Jewell and Brown, 2014; Smith et al., 2018) are also related to mental health and physical health challenges. Yet despite some common consequences, studies on discrimination due to race, gender, or weight have evolved as separate literatures, each examining negative consequences of particular stigmatized social identities in isolation.

But what if the target of racial discrimination is also the target of gender and weight discrimination? Consider, for example, the overweight Latinx girl who identifies as gender atypical and who reports discrimination or unfair treatment due to all of these devalued social identities (i.e., race/ethnicity, gender, and weight). Are her negative experiences exacerbated three-fold? Or are some types of discrimination particularly challenging independent of the presence of other social stigmas? In this example, just focusing on one stigmatized identity (ethnicity) ignores the fact that Latinx girls may confront the added challenges of gender and weight discrimination.

Researchers who study identity-based discrimination in adolescence are just beginning to simultaneously examine multiple types of discrimination. For example, Grollman (2012) asked adolescents from multiple racial/ethnic groups to report on the frequency of discrimination due to race, gender, sexual orientation, and socio-economic status (SES). Experiencing more types of discrimination was related to more self-reported depressive symptoms and physical health problems, which is consistent with the notion that people who possess multiple stigmatized identities are multiply disadvantaged (e.g., Beale, 1970; Meyer et al., 2008).

The research of (Grollman, 2012) took a variable-centered and additive approach to multiple stigmatized identities. Two recent studies have taken a person-centered approach to examine patterns of discrimination types that co-occur within groups of adolescents and the consequences of those patterns. Garnett et al. (2014) had multi-ethnic adolescents report on unfair treatment due to race, immigrant status, sexual orientation, and weight. Using latent class analysis, four classes or groups of respondents based on their patterns of perceiving discrimination due to these different identities were identified: a low discrimination class, race only class (high perceived discrimination due to race but low on the other identities), a sexual orientation class, and an intersectional class whose members reported discrimination due to race, immigrant status, and weight. Members of the intersectional class and the sexual orientation class reported the most emotional distress, suggesting that the nature of the stigmatized identity (sexual orientation) is just as meaningful as the number of identity-based discrimination types in predicting mental health outcomes. Using a similar analytic strategy and focusing on academic outcomes, Byrd and Andrews (2016) studied discrimination reported by middle and high school students due to race, gender, religion, SES, sexual orientation, and disability status, in addition to whether the source of the unfair treatment was peers or adults at school. Similar to Grollman (2012), the multiple stigmas class fared worse on perceived school climate and academic engagement.

We build on and extend this person-centered approach to multiple identity-based discrimination types in two important ways. First, no previous studies using these methods have examined the ethnic context as a predictor of different patterns of discrimination. Research on racial discrimination has documented that youth report more unfair treatment as the size of their racial/ethnic group is diminished (e.g., Benner and Graham, 2013). In school settings where most discrimination research takes place, adolescents may feel especially vulnerable to unfair treatment when there are few classmates who share their same race/ethnicity. Whether ethnic group size is a meaningful context variable for studying other identity-based types of discrimination is not yet known. Second, the source of perceived unfair treatment has also tended to be ignored, especially in research on stigmatized identities other than race. Does it make a difference, for example, if unfair treatment due to gender or weight is perpetrated by teachers versus peers at school? In the race/ethnicity literature, Benner and Graham (2013) found that unfair treatment from teachers (e.g., receiving a lower grade than you deserved) was related to academic disengagement whereas unfair treatment from peers (e.g., being excluded from the group) was linked to socioemotional challenges. It is likely that teacher discrimination will have a stronger impact on achievement than will peer discrimination because of the status and power of teachers to shape students’ academic outcomes. Peer discrimination, in contrast, may be particularly potent for socioemotional well-being given the importance of peer acceptance during adolescence.

We capitalized on a large and ethnically diverse sample of African American, Latinx, Asian, and White 8th grade students attending one of 26 middle schools that systematically varied in ethnic diversity and the numerical representation of each race/ethnic group. Participants reported on how much they experienced discrimination due to race/ethnicity, gender, and weight and whether the perpetrators of unfair treatment for each stigmatized identity were teachers or peers. We focused on race/ethnicity, gender, and weight because they are salient social identities among youth and all have been associated with discrimination during adolescence (Bucchianeri et al., 2013). Latent profile analysis (Lanza and Cooper, 2016), a person-centered approach, was used to identify patterns of perceived unfair treatment based on type and source of discrimination.

We addressed three research questions. First, do experiences of discrimination due to race/ethnicity, gender, and weight co-occur in the same individuals? If so, in what patterns? Second, what are the predictors of discrimination patterns? We included predictors relevant to particular discrimination types such as perceived gender atypicality and body mass index (BMI) as a measure of weight. We know that gender atypical and overweight youth lacking peer support may be at risk for peer-initiated unfair treatment (e.g., Juvonen et al., 2016; Smith et al., 2018), although it is less clear how these predictors map on to particular patterns of discrimination. Focusing on the ethnic context, we examined whether ethnic ingroup size influenced patterns of discrimination. We were particularly interested in whether smaller ethnic group size predicted more unfair treatment across patterns of discrimination. And third, we examined the academic, social, and mental health consequences of discrimination patterns. In contrast to the “more is worse” approach of most previous research, we asked whether particular patterns of co-occurring discrimination would map on to specific outcomes. In answering these questions, rather than testing specific hypotheses, our goal was to take a multiple identities perspective to achieve a more comprehensive, differentiated, and nuanced understanding of the experience of discrimination during early adolescence.

### Method

#### Participants

Participants were drawn from a larger sample of 4,702 eighth grade students participating in the UCLA Middle School Diversity Project. Students were enrolled in one of 26 middle schools in northern and southern California selected to represent a variety of ethnic compositions. Six schools were racially/ethnically diverse and members of each of the four major pan-racial/ethnic groups (i.e., African American, Asian, Latinx, and White) were fairly evenly represented; 9 were balanced schools, with two large and relatively equal-sized racial/ethnic groups (e.g., Asian and Latino); and 11 had a clear numerical majority racial/ethnic group (either African American, Asian, Latinx, or White) and smaller numbers from each of the other racial/ethnic groups. Based on student self-report, the sample was 33% Latinx, 21% White, 13% East or Southeast Asian, 11% African American, and 15% Multiethnic. The remaining 7% of the sample was comprised of students who self-reported as Middle Eastern, Pacific Islander, South Asian, or Other. Due to their small size and because most of these groups are not recognized as ethnic categories in school demographic data made available by the California Department of Education (CDE), they were excluded from the analyses. The final sample consisted of 4,574 participants (50% female; *M* = 14.04 years) with a racial/ethnic composition that was 43% Latinx, 28% White, 16% East or Southeast Asian, and 14% African American. About 75% of Latinx and Asian participants were second generation (at least one parent born outside of the United States).

To avoid confounding race/ethnicity and socioeconomic status (SES) in school selection, the sample was restricted to lower-middle and lower-SES communities. This was based on the percentage of students receiving free or reduced lunch and census data (e.g., median income, number of people in the work force) for neighborhoods in which schools were located. Of the 26 middle schools in this sample, 24 were eligible for Title I federal funds. Schools with average enrollments of 900–1200 students and reading and math achievement (40th to 60th percentile on standardized tests) were selected. The parent/guardian who completed informed consent at sixth grade (75% mothers) indicated their highest level of education (1 = elementary/junior high school, 2 = some high school, 3 = high school diploma or GED, 4 = some college, 5 = 4-year college degree, and 6 = graduate degree). Of the sample, 17% had less than a high school education, 11% received a high school diploma or GED, 25% had some college, 18% had a 4-year college degree, and 16% had a graduate degree. Table 2 indicates the breakdown of parent eduction by race/ethnicity. Although there was representation in each category for each racial/ethnic group, overall, African Americans were overrepresented in the some college category, Whites and Asians were overrepresented in the four-year college and graduate degree groups, and Latinxs’ were overrepresented in the less than high school categories.

**TABLE 2:** Parental education breakdown by race/ethnicity.

	Elementary/Junior high school	Some high school	High school diploma or GED	Some college	4-year college degree	Graduate degree
African American	2%	2%	10%	44%	20%	16%
Asian	7%	3%	10%	20%	26%	22%
Latino	25%	16%	18%	21%	9%	5%
White	1%	1%	4%	25%	31%	32%

#### Procedure

Surveys were group administered and read aloud by a trained graduate student researcher. Participants answered corresponding questions in survey booklets as a second trained research assistant circulated around the classroom to assist students as needed. On average, the survey took 1 h to complete, for which students received $10.

#### Measures

##### Peer and adult discrimination

Adolescents’ perceptions of peer discrimination at 8th grade were assessed using four items adapted from the Adolescent Discrimination Distress Index (ADDI, Fisher et al., 2000). A total of 12 items asked participants whether they had experienced exclusion, disrespectful treatment, threats, or name calling by their peers because of their race/ethnicity (e.g., “How often did kids exclude you from their activities because of your race/ethnic group?”), gender (e.g., “How often were you treated disrespectfully by other kids because of your gender?”), and weight (e.g., “How often were you called insulting names by other kids because of your weight?”). Another 12 items asked participants whether they had experienced unfair discipline, received a lower grade, others acting as though they were not smart, or disrespectful treatment by adults because of their ethnicity (e.g., “How often were you disciplined unfairly at school because of your ethnicity?”), gender (e.g., “How often were you given a lower grade than you deserved because of your gender?”), and weight (e.g., “How often were you treated disrespectfully by adults in your school because of your weight?”). Responses were rated on a 5-point scale, ranging from 0 (*never*) to 4 (*a whole lot*). The race/ethnicity discrimination subscales for peer and adult perpetrators show strong measurement invariance across multiple adolescent samples representing the racial/ethnic groups included in this study (Sladek et al., 2020). Given the positively skewed distribution of ratings (skewness_range_ = 1.53–8.98), with very few students reporting “*A lot*” and “*A whole lot*,” responses in both categories were collapsed with “*A few times*” to create a 3-point scale. This is in line with previous studies in the discrimination literature (Rivas-Drake et al., 2009; Benner and Graham, 2013).

##### Predictors of discrimination profiles

###### Gender

Students self-reported their gender. Gender was binary-coded, with females assigned values of 1 and males assigned values of 0.

###### Race/ethnicity

As in Study 1, race/ethnicity was based on self-report. Students were asked to select their race/ethnicity from the following options: American Indian, Black/African American, Black/other country of origin, East Asian, Latino, Mexican/Mexican American, Middle Eastern, Pacific Islander (including Filipino), South Asian, Southeast Asian, White/Caucasian, Multiethnic/Biracial, and Other. For the present study, some groups were combined (Black/African American and Black/other country of origin, East Asian and Southeast Asian, and Latino and Mexican/Mexican American). Race/ethnicity was dummy coded such that Whites were the reference group.

###### Body mass index

Body mass index was calculated from student self-reported age based on date of birth, height (“How tall are you?” __ feet __ inches), and weight (“How much do you weigh?” __ pounds) in 8th grade (*M* = 62.34; *SD* = 5.31). Self-reports have shown to be consistent with direct measures, and correlate with interviewer-measured reports (Goodman et al., 2000). The Centers for Disease Control (CDC) growth charts were used to calculate each individual’s BMI (kg/m^2^) percentile based on age and gender specific BMI percentiles (Kuczmarski et al., 2000).

###### Gender typicality

Unlike Study 1 that focused on perceived gender typicality of targets, our measure assessed self-perceived gender typicality. Adapted from a measure developed by Egan and Perry (2001), participants rated their agreement with three statements about whether they felt like a typical member of their gender group (e.g., “I feel like I am just like the other girls (boys) in my class”). Items were rated on a 5-point scale (from 1 = *not at all* to 5 = *all the time*) and were recoded such that higher means indicate greater perceived typicality (*α* = 0.76).

###### Proportion same race/ethnic peers in school

Complementing Study 1 that used a subjective measure of group size, we relied on objective school-level race/ethnicity data from the CDE website. CDE data were aggregated into four primary racial/ethnic categories: African American, Asian, Latinx, and White. Percent same-race/ethnicity peers reflects the proportion of same-grade students in the school that matched students’ racial/ethnic category. The values of this measure ranged from 0.01 to 0.67 for African Americans, 0.00 to 0.59 for Asians, 0.00 to 0.68 for Latinx, and 0.00 to 0.66 for Whites, indicating substantial differences in the relative size of each ethnic group across schools.

##### Outcome measures

###### Academic grade point average

Transcripts were obtained from school records and district data for each year of the study. First, grades of all academic core courses (English, math, science, social studies) were coded on a 12-point scale, ranging from 4.00 (A/A +) to 0.00 (F), with increments of 0.33 to indicate a grade that included a plus or minus. We then computed the average academic GPA by including the grades of all our study participants in each of these 8th grade core classes, (*M* = 2.80, *SD* = 1.01).

###### Depression

Depression was measured with 10 items from the Children’s Depression Inventory Short Form (CDI; Kovacs, 1992). Respondents were asked to choose the option that was most like how they were feeling in the past week (e.g., “I felt sad,” “I felt depressed”). Items were scored on a 4-point scale ranging from 1 (*rarely or none of the time-less than 1 day*) to 4 (*almost all the time – 5–7 days*) and were averaged with higher score indicating higher levels of depressive symptoms (*α* = 0.85).

###### Social anxiety

Social anxiety was measured using a shortened version of the Social Anxiety Scale for Adolescents (SAS-A; La Greca and Lopez, 1998). Items included “I worry about what other think of me” and were rated on a 5-point scale (1 = *Not at all* to 5 = *All the time*). A mean was computed with higher scores indicating greater social anxiety (*α* = 0.86).

###### Perceived coolness

Using a peer nomination procedure, participants selected as many students of either gender in their grade who fit different behavioral descriptions. To measure perceived coolness, participants nominated grademates who “are the coolest kids.” Coolness assesses characteristics related to power and visibility (notoriety) that classmates pay attention to and even admire (e.g., Kiefer and Wang, 2016). Having a reputation as cool is often used as a measure of high social status, particularly for adolescents (Closson, 2009). Nominations of coolness were standardized within gender and ranged from −0.73 to 10.93 (*M* = −0.73, *SD* = 0.97).

### Results

#### Analysis Plan

The analysis proceeded in two steps. First, we wanted to identify students with similar patterns of perceived discrimination from peers and adults based on three identities (race/ethnicity, gender, and weight) using latent profile analysis (LPA). LPA is a person-centered approach that identifies qualitatively distinct subgroups of youth with different discrimination experiences. Next we examined the predictors and consequences of particular profiles.

A planned missingness 3-form design (Graham et al., 2006) was implemented for the gender typicality, depression, and social anxiety measures. Procedures for handling missing data in LPAs using the 2-step BCH method are limited. However, because missingness based on planned missingness were assumed to be missing completely at random (MCAR), missing data was handled through listwise deletion.

#### What Is the Pattern of Discrimination Profiles?

We first fit a series of LPA models starting with a one-profile solution to a six-profile solution to explore the number and structure of latent subgroups to determine the best fitting LPA, including all correlates and outcomes as auxiliary variables. For model selection the Akaike information criterion (AIC), Bayesian information criterion (BIC), and sample adjusted BIC (ABIC) were used (Nylund et al., 2007). Lower values reflect better model fit. In addition to these fit statistics, classification quality (entropy value) and the conceptual clarity of the latent profiles in relation to theory and prior research were considered when comparing different class models. Entropy values greater than 0.80 indicate good classification accuracy (Reinecke, 2006).

Descriptive statistics and correlations among study variables are presented in Table 3. The 5-profile solution emerged as the best model for classifying discrimination profiles based on fit indices and conceptual interpretability (AIC = 82169.11; BIC = 82169.11; ABIC = 82615.51). The 5-profile solution was preferred because additional profiles represented only variations in profiles that had already been identified (see Meeus et al., 2010). The entropy value was 0.98.

**TABLE 3:** Intercorrelations, means, and standard deviations of all study variables.

	1	2	3	4	5	6	7	8	9	10	11	12	13
(1) Peer Gender Disc. Mean	–												
(2) Adult Gender Disc. Mean	0.403**	–											
(3) Peer Race Disc. Mean	0.459**	0.319**	–										
(4) Adult Race Disc. Mean	0.248**	0.509**	0.394**	–									
(5) Peer Weight Disc. Mean	0.367**	0.221**	0.375**	0.219**	–								
(6) Adult Weight Disc. Mean	0.300**	0.496**	0.265**	0.401**	0.427**	–							
(7) Gender Typ.	−0.245**	−0.092**	−0.147**	−0.099**	−0.199**	−0.121**	–						
(8) BMI	−0.004	0.022	−0.012	0.100**	0.192**	0.087**	−0.051*	–					
(9)% Same Ethnic Peers	−0.034*	0.000	−0.178**	−0.033	0.041*	0.024	−0.050*	0.057**	–				
(10) GPA	0.048**	−0.064**	0.024	−0.164**	−0.038*	−0.098**	0.016	−0.087**	0.095**	–			
(11) Depression	0.307**	0.190**	0.276**	0.208**	0.297**	0.143**	−0.289**	0.058**	−0.038	−0.031	–		
(12) Social Anxiety	0.228**	0.081**	0.272**	0.039	0.236**	0.115**	−0.217**	0.002	−0.025	0.131**	0.427**	–	
(13) Coolness	0.078**	0.110**	0.039*	0.087**	0.023	0.062**	−0.045	−0.029	0.023	−0.027	0.077**	−0.047*	–
**Mean**	0.21	0.11	0.39	0.24	0.28	0.05	3.30	3.30	0.4	2.80	1.68	2.06	−0.01
**SD**	0.39	0.32	0.52	0.46	0.49	0.22	1.00	1.00	0.18	1.01	0.64	0.74	0.97
**Range**	0−2	0−2	0−2	0−2	0−2	0−2	1−5	−5.91 to 3.1	0−0.68	0−4	1−4	1−5	−0.73 to 10.93
***N***	3626	3607	3625	3604	3626	3600	1851	3199	4239	3882	2301	1854	4463

Items for each discrimination subscale were averaged for the purposes of reporting descriptives, *p < 0.05. **p < 0.01. ***p < 0.001.

**FIGURE 4:**
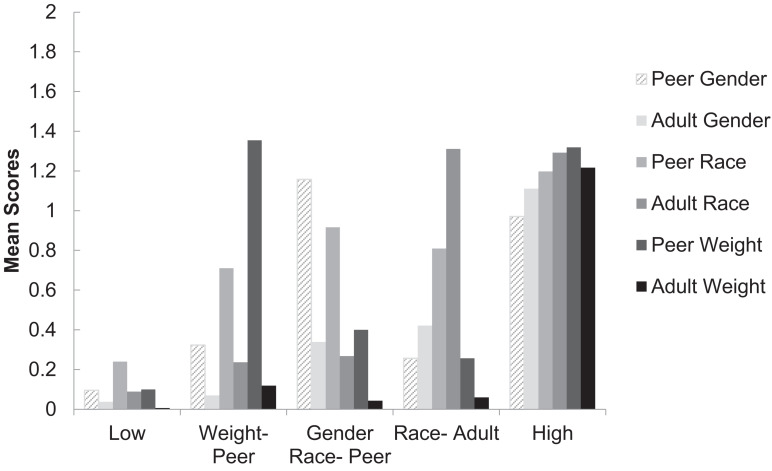
Discrimination profiles characterized by peer and adult discrimination across 3 stigmas (gender, race/ethnicity, and weight).

Figure 4 depicts the five profiles of discrimination. The largest profile type (74%, *n* = 3,119) consisted of adolescents who experienced relatively little to no discrimination across all three identities (race/ethnic group, gender, weight)—the *low discrimination* profile. The second largest profile (10%, *n* = 438) indicated a subgroup of youth who experienced relatively high weight-based discrimination from peers (*weight-peer discrimination*). The third profile (8%, *n* = 345) identified a subgroup of youth with relatively high levels of race-based discrimination from adults (*race/ethnicity-adult discrimination*). The fourth latent profile (5%, *n* = 189) indicated a subgroup of youth with relatively high levels of race/ethnicity- and gender-based discrimination mostly from peers (*race/gender-peer discrimination*). Finally, the smallest group (3%, *n* = 81) comprised a subgroup of youth who were relatively high in all types of discrimination from both peers and adults (*high discrimination*).

#### What Are the Predictors of Discrimination Profile Membership?

Next we examined differences in latent profile membership by gender, race/ethnicity, BMI, gender typicality, and proportion of same-race/ethnic peers in school (see Table 4). Because preliminary analyses showed no significant effect of parent education level, that variable was not included in the reported analyses. Models were fit using Mplus (Muthén and Muthén, 1998, version 7.3), and accounted for the clustering of youth within schools using the cluster function. All comparisons were made between the low discrimination profile as the reference group and all other profiles. Table 4 shows that the predictors differentially mapped on to specific profiles. Compared to the low discrimination profile, youth who were Asian and White, higher in BMI, and more gender atypical had an increased probability of being in the weight-peer discrimination profile. Unexpectedly, students were also more likely to be members of this profile when they attended middle schools with more same-ethnic peers. There were no significant differences in the probability of being in the weight-peer profile based on gender. As depicted in the second column of Table 3, White and African American girls who were more gender atypical were more likely to be in the race/gender-peer discrimination profile compared to the low discrimination profile. Turning to the third profile, youth who were male, African American or Latinx, higher in BMI, and more gender atypical had an increased probability of being in the race/ethnicity-adult discrimination profile. Consistent with previous research, membership in the race/ethnicity profile was also predicted by attending schools with fewer same race/ethnic peers. Finally, youth who were boys, higher in BMI, and more gender atypical had an increased probability of being in the high discrimination compared to the low discrimination profile.

**TABLE 4:** Predictors of discrimination profiles compared to low discrimination profile.

	Weight – Peer	Race Gender – Peer	Race – Adult	High
African American	−0.48^†^	−0.58	1.11**	−0.08
Asian	0.15	−0.92*	0.32	−0.31
Latino	−0.83**	−1.93***	1.30***	0.06
Gender	0.06	1.37***	−0.74**	−1.36*
BMI	0.02*	−0.01	0.01*	0.02*
Gender Typicality	−0.68**	−0.88***	−0.27**	−0.96***
% Same Ethnic Peers	2.23**	1.19	−1.48*	−0.98

#### What Outcomes Are Associated With Discrimination Profile Membership?

To examine outcomes of profiles, we used the Bolck–Croon–Hagenaars (BCH) approach (Bolck et al., 2004; Vermunt, 2010). This approach avoids shifts in profile membership when examining distal outcomes by using a weighted multiple group analysis reflecting the measurement error in the latent profile classifications, and by treating the profiles as known variables in the final stage (Asparouhov and Muthén, 2014; Bakk and Vermunt, 2016). We computed the profile-specific means and their standard errors of each academic, mental health, and peer status outcome and used a Wald chi-square test to examine significant differences between each profile, controlling for significant predictors (see Table 5). As in the analyses of predictors, particular profiles were differentially related to particular types of outcomes.

**TABLE 5:** Profile specific means for student outcomes.

	Low	Weight – Peer	Race Gender – Peer	Race – Adult	High
GPA	2.60^a^	2.68^a^	3.10^b^	2.05^c^	1.95^c^
Depression	1.56^a^	2.04^b^	2.17^b^	1.92^c^	2.15^b^
Social Anxiety	1.98^a^	2.40^b^	2.49^b^	2.03^a^	2.45^b^
Coolness	−0.05^a^	−0.06^a^	0.16^b^	0.24^b^	0.29^b^

*Row means with different superscripts are significantly different at the *p* < 0.05 level. Profile-specific mean differences were tested using Wald tests. Means were adjusted by controlling for all profile predictors.*

#### Achievement

Youth in the race/gender-peer profile had significantly higher GPAs (*M* = 3.10) compared to all other profiles. The low discrimination (*M* = 2.60) and weight-peer profiles (*M* = 2.68) also had relatively high GPAs, and were not significantly different from each other (Wald *χ2*(10) = 0.08, *p* = 0.57). The race/ethnicity-adult (*M* = 2.05) and high discrimination profiles (*M* = 1.95) had significantly lower GPAs than all other profiles, and were no different from each other (Wald *χ2*(10) = 0.10, *p* = 0.68).

#### Mental Health Outcomes

Youth in the low discrimination profile (*M* = 1.56) had significantly lower levels of depressive symptoms compared to all other profiles. They also had significantly lower levels of social anxiety (*M* = 1.98) compared to the other profiles except race/ethnicity-adult discrimination (*M* = 2.03; Wald *χ2*(10) = −0.04, *p* = 0.32). Of the profiles that experienced higher levels of discrimination, youth in the race/ethnicity-adult profile reported the least amount of depressive symptoms (*M* = 1.92) and social anxiety (*M* = 2.03), and were significantly lower in depressive symptoms and social anxiety than youth in the weight-peer (depression: *M* = 2.04, Wald *χ2*(10) = −0.12, *p* < 0.05; social anxiety: *M* = 2.40, Wald *χ2*(10) = −0.37, *p* < 0.001), high discrimination (depression: *M* = 2.15, Wald *χ2*(10) = −0.23, *p* < 0.05; social anxiety: *M* = 2.45, Wald *χ2*(10) = −0.42, *p* < 0.01), and race/gender-peer-profiles (depression: *M* = 2.17, Wald *χ2*(10) = −0.25, *p* < 0.01; social anxiety: *M* = 2.49, Wald *χ2*(10) = −0.47, *p* < 0.001). The weight-peer, race/gender-peer, and high discrimination profiles were not significantly different from each other. Thus, compared to the low discrimination group, the profiles with the best academic achievement (race/gender-peer, weight-peer) tended to report worse mental health outcomes.

#### Coolness

Some profiles were also differentially related to social standing among peers. Youth in the low discrimination (*M* = −0.05) and weight-peer discrimination profiles (*M* = −0.06) were perceived as the least cool by their peers. All comparisons between the low and weight-peer profiles were statistically significant except between the high discrimination profile (*M* = 0.29; low: Wald *χ2*(10) = 0.34, *p* = 0.07; weight-peer: Wald *χ2*(10) = 0.35, *p* = 0.05). Although youth in the high discrimination profile were perceived as the most cool, there was likely not enough power to detect the difference given this group is small in size. The race-gender/peer (*M* = 0.16), race/ethnicity-adult (*M* = 0.24), and high discrimination profile (*M* = 0.29) were perceived as cool by their peers, and were not significantly different from each other.

### Discussion

Findings from this study provide new insights into adolescents’ experiences with discrimination when viewed from a multiple identities perspective. Using a person-centered approach, we found distinct patterns of single and co-occurring discrimination due to race/ethnicity, gender, and weight from adults and peers at schools where participants’ racial/ethnic group size varied systematically. Most students (almost 75%) reported low levels of discrimination, which is consistent with other person-centered approaches (Garnett et al., 2014; Byrd and Andrews, 2016) as well as discrimination research in general (Benner et al., 2018). Self-report survey methods that dominate the field often yield low base rates. With this large sample, however, 25% reporting patterns of discrimination is meaningful and concerning. We documented a small group who reported experiencing all three types of unfair treatment from both sources. The remaining three groups were youth who had high probabilities of being in the peer weight discrimination, adult race/ethnicity, and peer race/ethnicity-gender discrimination profiles. Examining predictors and consequences of these profiles provided the more nuanced and differentiated understanding of what it means to feel multiple types of identity-based discrimination during early adolescence.

One predictor of discrimination that was similar across all profiles was gender typicality. As youth perceived themselves to be different from their peers on normative gender behavior, they felt more mistreated. A growing literature documents a strong relationship between gender atypicality and experiences of bullying during the early adolescent years (e.g., Jewell and Brown, 2014; Smith et al., 2018). Perceived deviation from gender norms during middle school may therefore be a potent risk factor for maladjustment that cuts across multiple stigmatized identities. However, we remain cautious in this interpretation because there are many contextual factors not examined here that influence the relation between gender atypicality and psychosocial adjustment. For example Smith and Leaper (2005) found that gender non-conforming adolescents in their sample did not report adjustment difficulties (i.e., low self-worth) if they were accepted by peers and did not feel pressure from them to conform. Future research should continue to unpackage how the social context shapes the extent to which perceived gender atypicality is a risk factor for later maladjustment.

Considering other predictors that varied across profiles, some of the most informative findings emerged when examining the race/ethnicity-adult and weight-peer profiles specifically. Discrimination due to race/ethnicity from adults captured respondents who perceived that teachers graded and disciplined unfairly and generally treated them in a disrespectful manner. Not surprisingly, being bigger (higher BMI scores), male, and African American or Latinx predicted membership in this profile. Ethnic minority boys from marginalized groups are often stereotyped as academically disengaged and aggressive and they are observed to have more conflict with teachers (e.g., Howard, 2014). Research also documents that African American boys in particular are perceived to be bigger and older than they actually are, which contributes to the stereotype associating being Black and male with violent intent (Goff et al., 2014). Our school context predictor also revealed that youth had a higher probability of membership in the race/ethnicity-adult profile as the size of their own racial/ethnic group declined. Having a critical mass of school mates who look “like me” appears crucial for minority males with marginalized identities to ward off the perception that teachers treat them unfairly. While our findings highlight the plight of ethnic minority boys, we do not want to lose sight of the fact that ethnic minority girls are also vulnerable to such marginalization. Pervasive media portrayals of Black girls as angry and loud (e.g., *Sapphire*) or hypersexualized (e.g., *Jezebel*) and Latinx girls as sexually promiscuous and gang-affiliated (e.g., *cholas*) contribute to stereotypes intersecting gender and race/ethnicity that also impact perceived and actual unfair treatment by adults (Blake et al., 2011; Lopez and Chesney-Lind, 2014; Morris, 2016).

Predictors of weight discrimination from peers were quite different. This profile had a greater probability of members who were White and Asian with larger BMIs, and who attended schools with more same-race peers. According to national trends, White and Asian youth are lower on overall BMI scores than African American and Latinx youth (Ogden et al., 2016). Thus, an overweight Asian or White adolescent likely deviates from the weight norm for their racial group. When there are many same-race peers in one’s school, this deviation is even greater. Previous research has documented that being different (stigmatized) on an important social identity such as weight status promoted biased-based bullying, a particular form of discrimination (Juvonen et al., 2016; Lanza et al., 2018). If the stigma associated with being overweight is greater in societally more privileged racial groups, being surrounded by more same-race peers who see you as different can be a risk factor. For marginalized groups, in contrast, who perceive unfair treatment from powerful others due to their race or ethnicity, the *absence* of more ingroup members who share your identity is the risk factor. Thus the relative size of one’s racial/ethnic group can be a risk or protective factor depending on how gender, race/ethnicity, and weight interact to predict unfair treatment and whether the source of that mistreatment is teachers or peers. We believe that the study of discrimination during adolescence needs greater attention to the complexities of ingroup representation across different racial/ethnic groups and the way it shapes the likelihood that a social identity will be perceived as stigmatized.

We also documented a systematic mapping of particular consequences on specific discrimination profiles, but one more complex than the “more is worse” pattern in previous studies of multiple identities. In our research, membership in profiles where adults at school were the perceived perpetrators (the race/ethnicity and high groups) was associated with lower GPA. Yet these same African American and Latinx boys were also perceived as most cool by their grademates. Coolness capturs one’s position in the social hierarchy that middle school students are particularly anxious to climb (e.g., Kiefer and Wang, 2016) and what constitutes coolness is shaped by the particular school context and configuration of racial/ethnic groups (Way et al., 2008). Perhaps ethnic minority boys achieve some short-term notoriety when they disengage at the beginning of middle school as a result of perceived unfair treatment by teachers (e.g., Graham et al., 1998). Longitudinal research will need to examine the cumulative tradeoff between low achievement accompanied by high social standing (e.g., Yun and Graham, 2019).

In contrast, membership in profiles associated with peer discrimination (weight, race/gender) was correlated with psychosocial challenges as measured by depressive symptoms and social anxiety. Early adolescence is a period when youth are especially motivated to find their niche and “fit in” (e.g., Eccles and Roeser, 2011); thus, experiencing unfair treatment by peers such as being excluded from the group or called bad names will be psychologically painful. Yet these same profile members – White and Asian boys and girls, African American girls – enjoyed overall higher GPAs which, we suggest, mitigated their psychosocial maladjustment. Similarly, having a reputation as cool and fewer mental health challenges may have modulated the effects of relatively lower achievement for African American and Latinx boys. Feeling bad and doing poorly in school are two well-documented effects of perceived discrimination during adolescence (Benner et al., 2018). They do not always go hand-in-hand once we take into account the race/ethnicity and gender of participants and how these characteristics intersect with multiple identities that can be stigmatizing depending on context.

## General Discussion

In two studies, we demonstrated the importance of examining multiple identities as well as the context in which these identities operate for two key dimensions of intergroup relations – perception and experiences. Using intersectionality and multiple identities approaches, each study provided unique insights into how the racial/ethnic context –whether perceived or actual—can change the meaning and significance of an identity and the ways in which it works together with other identities to shape perception, experiences, and outcomes. To our knowledge, we are the first to simultaneously examine the role of numerical size as the racial/ethnic context in how social categories fuse (intersectionality) and configure (multiple identities) and implications for intergroup attitudes, well-being, and academic achievement.

On the *perception* side of our work (Study 1), the simultaneous consideration of multiple social categories and perceived group size allowed for the identification of which combination of categories most strongly influence intergroup attitudes. We found that gender and sexual orientation (but not race/ethnicity) drove African American and Latinx respondents’ gender typicality stereotypes, intergroup emotions and behavioral tendencies. Consistent with an intersectional perspective perceived group size changed the meaning and significance of identity intersections for intergroup emotions and behavioral tendencies. Group size, however, did not affect gender typicality ratings of lesbian and gay FB profiles. On the *experience* side of our work (Study 2), we demonstrated that youths’ reports of identity-based unfair treatment are complex. Consistent with the multiple identity approach, different forms of discrimination co-occur albeit with different levels of prominence. This contextualized and person-centered approach can better capture the complexity and heterogeneity of identity-based discrimination experiences and its implications for outcomes.

Our studies make several important theoretical and methodological contributions. Theoretically, our work shows the value of simultaneously considering multiple identities using intersectionality and multiple identities frameworks. These frameworks were originally developed in research with adults. Yet an understanding of how individuals’ many identities are interrelated and affect outcomes is a gradual developmental process (Azmitia et al., 2008). Thus, taking a developmental perspective can shed light on the process by which intersectional identities and social locations affect people’s lives. To that end, we situate our work among early adolescents –young people at a critical developmental period that allow for emergent understandings of systems of power, privilege, and disadvantage that give meaning to status-based identities and experiences.

Our work also makes methodological contributions. We examined the ways in which early adolescents make sense of the embedded systems of power and their own and others’ location in these intersectional spaces. We operationalized *one* aspect of the systems embedded in youths’ context –namely the size of their own racial/ethnic group. Because race/ethnicity is a prominent feature of urban schools along which youth often sort themselves, focusing on the presence of same-race/ethnicity peers is important. Nevertheless, we recognize that the school ethnic context can be defined in ways other than group size such as schools’ valuing of multiculturalism (e.g., Brown and Chu, 2012). We used perceived and actual presence of same-race/ethnicity peers to operationalize group size, and by extension, power and status of that group. Each indicator provides distinct information. In Study 1, we showed that perceived group size changed the pattern of intersectional bias. In Study 2, actual group size affected which group of youth experienced which constellation of identity-based unfair treatment. While youths’ *perception* of group size can be cognitively or motivationally biased, showing under-or over-estimation, the *actual* group size provides an unbiased assessment system over and above what the individual perceives. Future research will benefit from examining the implications of the joint impact of both subjective and objective group size on intersectional intergroup bias and outcomes.

The two studies together also highlight the value of disentangling societal and numerical status. Because the numerical majority racial/ethnic group in most prior research with adults on intergroup dynamics was typically White and the numerically smaller group was typically Black, larger group size has often been linked to power, privilege, and advantage whereas smaller group size has frequently been associated with disadvantage (e.g., Hewstone et al., 2002). Our sampling strategy allowed us to independently assess race/ethnicity and numerical status (perceived and actual). Being a numerical racial/ethnic majority was associated with power even among societal low status groups (Latinx and African American) in Study 1 whereas being a numerical majority was associated with weight discrimination risk among societal high status groups (White and Asian) in Study 2. Depending on context, then, numerical minority status need not be synonymous with low status just as numerical majority status does not guarantee power and prestige. Future research should further disentangle racial/ethnic group size and status across different domains and guard against research questions that conflate the two.

As researchers begin to incorporate more than one identity in developmental science research, creating age-appropriate and meaningful methods is critical. The current research, for example, demonstrated the value of an intersectionality approach through the use of Facebook-like profiles. Using that platform, we were able to specify multiple social categories such as gender, race/ethnicity, and sexual orientation simultaneously and in an organic, naturalistic way. Because most adolescents know about Facebook (e.g., Yang and Brown, 2013) and about half of 13–17 year olds use it (Pew Research Center, 2018), the Facebook format can provide a novel way to assess adolescents’ intersectional intergroup attitudes. Nevertheless, a recent survey conducted by the Pew Research Center (2018) documents that youths today use a wider range of social media such as YouTube, Instagram or Snapchat –platforms that have unique set ups different from Facebook. Newer social media platforms provide exciting opportunities for researchers to operationalize various intersectional identities and experiences in future studies.

While most early psychological intersectionality research used qualitative methods, more recent studies have incorporated quantitative approaches (Mays and Ghavami, 2018). By using surveys, we, too, assessed perception and experiences of intergroup group dynamics with a quantitative methodology. We asked parallel questions about discrimination related to different identities (Study 2) and asked the same questions about stereotypes, emotion, and behavioral tendencies after adolescents viewed different race/ethnicity, gender and sexual orientation FB profiles (Study 1). A main assumption of this quantitative approach is that intergroup experiences differ among groups in magnitude but not in content. As a case in point, we document in Study 2 that higher BMI White and Asian girls were in the weight profile because they reported high frequency ratings on items assessing weight discrimination from peers relative to the other types of discrimination. Based on these frequency data alone, we cannot determine whether and how the content of weight-based discrimination is the same or different for overweight boys and girls who are White or Asian. For example, does being treated unfairly because you are heavier than your peers mean the same thing for Asian girls compared to White boys or even for all Asian girls? In a study of microaggressions reported by Black women, Lewis and Neville (2015) identified multiple categories of everyday unfair treatment such as negative comments about physical appearance, silencing techniques, and inappropriate assumptions about behavioral or emotional style. These authors concluded that the discrimination experiences of Black women at the intersection of race and gender are quite unique. We cannot know with our methods how the content of gendered racial/ethnic discrimination experiences vary for different groups. Thus the richness of conceptualizing discrimination as experienced at individual intersections is not captured. On the other hand, we must be cautious about focusing too much on intersection-specific discrimination with ethnically diverse samples because such analyses can become unwieldy and artificially restrict the full range of discrimination experiences (see Bauer and Scheim, 2019). The tradeoffs of focusing on magnitude versus content in multiple identities and intersectionality research has important conceptual and methodological challenges that should be addressed in future research.

### Strengths and Limitations

The studies reported here focused on how the racial/ethnic context worked together with social identities to shape intergroup perception, experiences, and outcomes. A strength of both studies was the use of a large ethnically diverse sample of middle school students, which permitted an examination of many combinations of gender, race/ethnicity, weight, and sexual orientation. Another strength of this research, especially of Study 2, was sampling students from a large number of ethnically diverse urban middle schools with different combinations of racial/ethnic groups and the relative representation of each. Yet by highlighting ethnic ingroup size, we did not take into account the configurations of other racial/ethnic groups that comprised any school’s student population. An important direction for future research would be to examine not only the size of one’s own racial/ethnic group in school but also the other race/ethnicities with whom any one group co-exists and how these configurations may have played a role in shaping intergroup attitudes and outcomes. In addition, because of the racial/ethnic composition of the school district from which Study 1 participants were drawn, for example, Latinx students generally attended schools in which they were the numerical majority. This was reflected in our sample where Latinx were the majority in all four schools whereas African American students were consistently the minority in each school. Even though participants varied in their perception of group size, there was no opportunity to observe intergroup attitudes of, for example, Latinx students in schools with very low proportions, or African American students in schools with very high proportions of same-race/ethnicity peers.

Another limitation is that our studies are cross-sectional. Some longitudinal research indicates that perceptions of the supportiveness of teachers and peers decline across the middle school years (Way et al., 2007) and that experiences with racial discrimination increase for many youth (e.g., Smith-Bynum et al., 2014). In the absence of more longitudinal research, we cannot determine when and how the patterns documented here emerge and when they may begin to shift as a function of age and other developmental and contextual changes. Finally, because of the small sample size of sexual minority youth and the complex design of Study 1, we focused only on youth who self-identified as heterosexual. It would be informative to examine whether the intergroup attitudes held by LGB adolescents are similar to or different from those held by heterosexual adolescents.

### Implications for Intervention

The perceivers of Study 1 – if they endorse stereotypes based on intersectional identities or distance themselves emotionally and behaviorally – could be the peer perpetrators of perceived discrimination in Study 2. Taken together, the findings of both studies therefore have implications for intervention to address the plight of multiply stigmatized youth. It is evident to us that a single identity approach will not be adequate. Stigmatized identities intersect and co-occur; they have different effects in different domains across different levels of the racial/ethnic context. For example, if the interventionist’s goal is to reduce the impact of identity-based discrimination, then it will be important to specify the identities in question and then focus on the outcomes most directly linked to (predicted by) the combination of those identities. For ethnic minority males in the race/ethnicity profile (constellation of identities where race/ethnicity is relatively more prominent), it may not be reasonable to focus on mental health outcomes if they are not compromised, just as achievement strivings may be less of an issue for youth who experience the constellation of weight-based discrimination.

On the perceiver side, most approaches to improving intergroup relations focus on conditions of contact (e.g., Allport, 1954) but based on a single identity, such as race or sexual orientation. In addition, these interventions have primarily targeted intergroup majority/minority relations (Whites vs African Americans) rather than intragroup dynamics (straight African American boys vs gay African American boys) (Pettigrew and Tropp, 2006). Finally, these efforts have generally left out the role of structural and contextual influences. Our work highlights the need for a targeted and contextualized approach to reducing bias, given that youth, especially gay boys, in racially homogeneous contexts may be at an especially high risk for rejection. Consistent with our findings, an early study of racial/ethnic minority LGBQ middle and high school students documented a greater risk of victimization of these youth in schools where their racial/ethnic group was in the majority (Kosciw, 2004). Interventions at the structural level should therefore promote racially/ethnically diverse schools (no one group is in the numerical majority) so that multiply marginalized youth are exposed to different groups and different norms. These exposures can provide opportunities for youth to find their niche and fit in.

## Conclusion

Today’s urban schools provide a unique intergroup context, one where the student body varies not only based on race/ethnicity but also based on the relative representation of each racial/ethnic group. This diversity is likely to work together with social identities such as race/ethnicity, gender, and sexual orientation to shape how students are perceived and responded to, as well as how they feel about themselves. Our work contributes to the changing landscape of education by demonstrating that youths’ experiences are complex and that these experiences extend far beyond what a single axis or a decontextualized approach can capture. Intersectional and multiple identities approaches make it clear that to focus on *either* race/ethnicity, gender, weight, *or* sexual orientation limits understanding of adolescents’ intergroup perceptions and experiences. The consequences of intersecting social categories are complex and depend on the domain and the diversity of context. Thus, to understand developmental intergroup dynamics, we need to consider how social identities work together in context.

## Data Availability Statement

The raw data supporting the conclusions of this article will be made available by the authors, without undue reservation, to any qualified researcher.

## Ethics Statement

The studies involving human participants were reviewed and approved by University of California, Los Angeles. Written informed consent to participate in this study was provided by the participants’ legal guardian/next of kin.

## Author Contributions

NG and SG conceptualized the ideas for the manuscript. NG collected, analyzed, and wrote the findings as well as the introduction and discussion for Study 1. KK and SG planned and analyzed the data for Study 2 and wrote the findings well as the introduction and discussion of that study. NG and SG wrote the general introduction and discussion of the manuscript. All authors contributed to the article and approved the submitted version.

## Conflict of Interest

The authors declare that the research was conducted in the absence of any commercial or financial relationships that could be construed as a potential conflict of interest.

## Footnotes

1 We recognize that the ethnic label “Latino/Latina” reflects a gender binary and that some scholars have moved toward using the gender inclusive label “Latinx.” Because this study is part of a larger project, one in which comparisons with racial/ethnic data gathered from both the United States and California Departments of Education is a goal, participants were provided with the Latino/a label.

2 Title 1 funding provides government financial assistance to schools with high numbers or high percentages of youth from low-income families to help ensure that youth meet state academic standards.

## References

[B1] Akiba D. Szalacha L. A. García Coll C. T. (2004). Multiplicity of ethnic identification during middle childhood: conceptual and methodological considerations. *New Directions Child Adolescent Dev.* 2004 45–60. 10.1002/cd.103 15283078

[B2] Allport G. W. (1954). *The Nature of Prejudice.* Boston, MA: Addison-Wesley.

[B3] Ashmore R. D. Deaux K. McLaughlin-Volpe T. (2004). An organizing framework for collective identity: articulation and significance of multidimensionality. *Psychol. Bull.* 130 80–114. 10.1037/0033-2909.130.1.80 14717651

[B4] Asparouhov T. Muthén B. (2014). Auxiliary variables in mixture modeling: using the BCH method in Mplus to estimate a distal outcome model and an arbitrary secondary model. *Mplus Web Notes* 21 1–22.

[B5] Azmitia M. Syed M. Radmacher K. (2008). On the intersection of personal and social identities: introduction and evidence from a longitudinal study of emerging adults. *New Directions Child Adolescent Dev.* 2008 1–16. 10.1002/cd.212 18521867

[B6] Bakk Z. Vermunt J. K. (2016). Robustness of stepwise latent class modeling with continuous distal outcomes. *Struct. Equ. Model. Multidiscip. J.* 23 20–31. 10.1080/10705511.2014.955104

[B7] Bauer G. R. Scheim A. I. (2019). Methods for analytic intercategorical intersectionality in quantitative research: discrimination as a mediator of health inequalities. *Social Sci. Med.* 226 236–245. 10.1016/j.socscimed.2018.12.015 30674435

[B8] Beale F. (1970). “Double jeopardy: to be black and female,” in *The Black Woman*, ed. Cade T. (New York, NY: New American Library), 90–100.

[B9] Benner A. Graham S. (2013). The antecedents and consequences of racial/ethnic discrimination during adolescence: does the source of discrimination matter? *Dev. Psychol.* 49 1602–1613. 23106845 10.1037/a0030557

[B10] Benner A. Wang Y. Shen Y. Boyle A. Polk R. Cheng Y. (2018). Racial/ethnic discrimination and well-being during adolescence: a meta-analytic review. *Am. Psychol.* 73 855–883. 10.1037/amp0000204 30024216 PMC6172152

[B11] Bettencourt B. A. Bartholow B. D. (1998). The importance of status legitimacy for intergroup attitudes among numerical minorities. *J. Social Issues* 54 759–775. 10.1111/j.1540-4560.1998.tb01247.x

[B12] Bigler R. S. Liben L. S. (2007). Developmental intergroup theory: explaining and reducing children’s social stereotyping and prejudice. *Curr. Direct. Psychol. Sci.* 16 162–166. 10.1111/j.1467-8721.2007.00496.x

[B13] Blake J. J. Butler B. R. Lewis C. W. Darensbourg A. (2011). Unmasking the inequitable discipline experiences of urban Black girls: implications for urban educational stakeholders. *Urban Rev.* 43 90–106. 10.1007/s11256-009-0148-8

[B14] Bolck A. Croon M. Hagenaars J. (2004). Estimating latent structure models with categorical variables: one-step versus three-step estimators. *Political Anal.* 12 3–27. 10.1093/pam/mph001

[B15] Bond J. C. Perry P. (1970). “Is the black male castrated?,” in *The Black Women*, ed. Cade T. (New York, NY: New American Library), 115–118.

[B16] Brown C. S. Chu H. (2012). Discrimination, ethnc identity, and academic outcomes of Mexican immigrant children: the importance of school context. *Child Dev.* 83 1477–1485. 10.1111/j.1467-8624.2012.01786.x 22966916

[B17] Brown C. S. (ed). (2017). *Discrimination in Childhood and Adolescence: Patterns Across Social Groups.* Milton: Routledge/Taylor & Francis.

[B18] Bucchianeri M. Eisenberg M. Neumark-Sztainer D. (2013). Weightism, racism, classism, and sexism: shared forms of harassment in adolescents. *J. Adolescent Health* 53 47–53. 10.1016/j.jadohealth.2013.01.006 23566562 PMC3691304

[B19] Byrd C. Andrews D. (2016). Variations in students’ perceived reasons for, sources of, and forms of in-school discrimination: a latent class analysis. *J. School Psychol.* 57 1–14. 10.1016/j.jsp.2016.05.001 27425562

[B20] Chen X. Graham S. (2015). Cross−ethnic friendships and intergroup attitudes among Asian American adolescents. *Child Dev.* 86 749–764. 10.1111/cdev.12339 25626492 PMC4428968

[B21] Closson L. (2009). Status and gender differences in early adolescents’ descriptions of popularity. *Social Dev.* 18 412–426. 10.1111/j.1467-9507.2008.00459.x

[B22] Cole E. R. (2009). Intersectionality and research in psychology. *Am. Psychol.* 64 170–180. 10.1037/a0014564 19348518

[B23] Collins R. (2000). Situational stratification: a micro-macro theory of inequality. *Sociol. Theory* 18 17–43. 10.1111/0735-2751.00086

[B24] Crenshaw K. W. (1991). Mapping the margins: intersectionality, identity politics, and violence against women of color. *Stanford Law Review* 43 1241–1299. 10.2307/122903

[B25] Crenshaw K. W. (1995). “Mapping the margins: intersectionality, identity politics, and violence against women of color,” in *Critical Race Theory: The Key Writings that Formed the Movement*, eds Crenshaw K. W. Gotanda N. Peller G. Thomas K. (New York, NY: New Press), 357–384.

[B26] Crocker J. Major B. Steele C. (1998). “Social stigma,” in *The Handbook of Social Psychology*, Vol. 2 eds Gilbert D. Fiske S. Lindzey G. (New York, NY: McGraw-Hill), 504–553.

[B27] Cross W. E. Jr. (1991). *Shades of Black: Diversity in African-American Identity.* Philadelphia, PA: Temple University Press.

[B28] Cuddy A. J. C. Fiske S. T. Glick P. (2007). The BIAS map: behaviors from intergroup affect and stereotypes. *J. Pers. Social Psychol.* 92 631–648. 10.1037/0022-3514.92.4.631 17469949

[B29] Eccles J. S. Roeser R. W. (2011). “School and community influences on human development,” in *Developmental Science: An Advanced Textbook*, eds Bornstein M. H. Lamb M. E. (East Sussex: Psychology Press), 571–643.

[B30] Egan S. K. Perry D. G. (2001). Gender identity: a multidimensional analysis with implications for psychosocial adjustment. *Dev. Psychol.* 37 451–463. 10.1037/0012-1649.37.4.451 11444482

[B31] Erikson E. H. (1968). *Identity: Youth and Crisis.* New York, NY: W.W. Norton & Company.

[B32] Espinoza G. Gonzales N. A. Fuligni A. J. (2016). Parent discrimination predicts Mexican−American adolescent psychological adjustment 1 year later. *Child Dev.* 87 1079–1089. 10.1111/cdev.12521 27224903 PMC4939107

[B33] Fisher C. B. Wallace S. A. Fenton R. E. (2000). Discrimination distress during adolescence. *J. Youth Adolescence* 29 679–695. 10.1023/A:1026455906512

[B34] Garcia-Coll C. Crnic K. Lamberty G. Wasik B. H. Jenkins R. Garcia H. V. (1996). An integrative model for the study of developmental competencies in minority children. *Child Dev.* 67 1891–1914. 10.1111/j.1467-8624.1996.tb01834.x 9022222

[B35] Garnett B. Mssyn K. Austin A. Miller M. Williams S. Viswanath K. (2014). The intersectionality of discrimination attributes and bullying among youth: an applied latent class analysis. *J. Youth Adolescence* 43 1225–1239. 10.1007/s10964-013-0073-8 24318776

[B36] Ghavami N. Mistry R. S. (2019). Urban ethnically diverse adolescents’ perceptions of social class at the intersection of race, gender, and sexual orientation. *Dev. Psychol.* 55 457–470. 10.1037/dev0000572 30802098

[B37] Ghavami N. Peplau L. A. (2018). Urban middle school students’ stereotypes at the intersection of ethnicity, sexual orientation and gender. *Child Dev.* 89 881–896. 10.1111/cdev.12763 28262919

[B38] Goff P. Jackson M. Allison B. DiLeone L. Culotta C. DiTomasso N. (2014). The essence of innocence: consequences of dehumanizing Black children. *J. Pers. Soc. Psychol.* 106 526–545. 10.1037/a0035663 24564373

[B39] Goodman E. Hinden B. R. Khandelwal S. (2000). Accuracy of teen and parental reports of obesity and body mass index. *Pediatrics* 106 52–58. 10.1542/peds.106.1.52 10878149

[B40] Graham J. W. Taylor B. J. Olchowski A. E. Cumsille P. E. (2006). Planned missing data designs in psychological research. *Psychol. Methods* 11 323–343. 10.1037/1082-989X.11.4.323 17154750

[B41] Graham S. (2018). Race/ethnicity and social adjustment of adolescents: how (not if) school diversity matters. *Educ. Psychol.* 53 64–77. 10.1080/00461520.2018.1428805

[B42] Graham S. Taylor A. Z. Hudley C. (1998). Exploring achievement values among ethnic minority early adolescents. *J. Educ. Psychol.* 90 606–620. 10.1037/0022-0663.90.4.606

[B43] Grollman E. A. (2012). Multiple forms of perceived discrimination and health among adolescents and young adults. *J. Health Soc. Behav.* 53 199–214. 10.1177/0022146512444289 22588219

[B44] Grollman E. A. (2014). Multiple disadvantaged statuses and health: the role of multiple forms of discrimination. *J. Health Social Behav.* 55 3–19. 10.1177/0022146514521215 24578393

[B45] Hewstone M. Rubin M. Willis H. (2002). Intergroup bias. *Annu. Rev. Psychol.* 53 575–604. 10.1146/annurev.psych.53.100901.135109 11752497

[B46] Howard T. C. (2014). *Black Male(d): Peril and Promise in the Education of African American Males.* New York, NY: Teachers College Press.

[B47] IBM Corp (2013). *Released 2013. IBM SPSS Statistics for Windows, Version 24.0.* Armonk, NY: IBM Corp.

[B48] Jaret C. Reitzes D. C. (1999). The importance of racial-ethnic identity and social setting for blacks, whites, and multiracials. *Sociol. Perspect.* 42 711–737. 10.2307/1389581

[B49] Jewell J. Brown C. (2014). Relations among gender typicality, peer relations, and mental health during early adolescence. *Social Dev.* 23 137–156. 10.1111/sode.12042

[B50] Juvonen J. Lessard L. M. Schacter H. L. Suchilt L. (2016). Emotional implications of weight stigma across middle school: the role of weight-based peer discrimination. *J. Clin. Child Adolescent Psychol.* 46 150–158. 10.1080/15374416.2016.1188703 27617887 PMC6105282

[B51] Juvonen J. Nishina A. Graham S. (2006). Ethnic diversity and perceptions of safety in urban middle schools. *Psychol. Sci.* 17 393–400. 10.1111/j.1467-9280.2006.01718.x 16683926

[B52] Kiang L. Yip T. Fuligni A. J. (2008). Multiple social identities and adjustment in young adults from ethnically diverse backgrounds. *J. Res. Adolescence* 18 643–670. 10.1111/j.1532-7795.2008.00575.x

[B53] Kiefer S. Wang J. (2016). Associations of coolness and social goals with aggression and engagement during adolescence. *J. Appl. Dev. Psychol.* 44 52–62. 10.1016/j.appdev.2016.02.007

[B54] Kite M. E. Deaux K. (1987). Gender belief systems: homosexuality and the implicit inversion theory. *Psychol. Women Q.* 11 83–96. 10.1111/j.1471-6402.1987.tb00776.x

[B55] Kogachi K. Graham S. (2020). Numerical minority status in middle school and racial/ethnic segregation in academic classes. *Child Dev.* 10.1111/cdev.13408 33460066

[B56] Kosciw J. G. (2004). *The 2003 National School Climate Survey. The School-Related Experiences of Our Nation’s Lesbian, Gay, Bisexual and Transgender Youth.* New York, NY: GLSEN (Gay, Lesbian and Straight Education Network).

[B57] Kovacs M. (1992). *Children’s Depression Inventory.* North Tonawanda: Multi-Health Systems.

[B58] Kuczmarski R. J. Ogden C. L. Grummer-Strawn L. M. Flegal K. M. Guo S. S. Wei R. (2000). *CDC Growth Charts: United States Advance Data from Vital and Health Statistics, no. 314.* Hyattsville, MD: National Center for Health Statistics.

[B59] La Greca A. M. Lopez N. (1998). Social anxiety among adolescents: linkages with peer relations and friendships. *J. Abnormal Child Psychol.* 26 83–94. 10.1023/A:1022684520514 9634131

[B60] Lanza H. I. Echols L. Graham S. (2018). A silver lining: the role of ethnic diversity on co-occurring trajectories of weight status and peer victimization across early adolescence. *J. Adolescent Health* 63 554–560. 10.1016/j.jadohealth.2018.05.026 30170938 PMC6752051

[B61] Lanza S. T. Cooper B. R. (2016). Latent class analysis for developmental research. *Child Dev. Perspect.* 10 59–64. 10.1111/cdep.12163 31844424 PMC6914261

[B62] Lewis J. A. Neville H. A. (2015). Construction and initial validation of the Gendered Racial Microaggressions Scale for Black women. *J. Counseling Psychol.* 62 289–302. 10.1037/cou0000062 25867696

[B63] Liben L. S. (2014). The Individual ? Context nexus in developmental intergroup theory: within and beyond the ivory tower. *Res. Human Dev.* 11 273–290. 10.1080/15427609.2014.967048

[B64] Lopez V. Chesney-Lind M. (2014). Latina girls speak out: stereotypes, gender, and relationship dynamics. *Latino Stud.* 12 527–549. 10.1057/lst.2014.54

[B65] Mays V. M. Ghavami N. (2018). “History, aspirations, and transformations of intersectionality: focusing on gender,” in *APA Handbooks in Psychology§. APA Handbook of the Psychology of Women: History, Theory, and Battlegrounds*, eds Travis C. B. White J. W. Rutherford A. Williams W. S. Cook S. L. Wyche K. F. (Washington, DC: American Psychological Association), 541–566. 10.1037/0000059-028

[B66] Meeus W. Van De Schoot R. Keijsers L. Schwartz S. J. Branje S. (2010). On the progression and stability of adolescent identity formation: a five−wave longitudinal study in early−to−middle and middle−to−late adolescence. *Child Dev.* 81 1565–1581. 10.1111/j.1467-8624.2010.01492.x 20840241

[B67] Meyer I. H. (2003). Prejudice, social stress and mental health in lesbian, gay and bisexual populations: conceptual issues and research evidence. *Psychol. Bull.* 129 674–697. 10.1037/0033-2909.129.5.674 12956539 PMC2072932

[B68] Meyer I. H. Dietrich J. Schwartz S. (2008). Lifetime prevalence of mental disorders and suicide attempts in diverse lesbian, gay, and bisexual populations. *Am. J. Public Health Res.* 98 1004–1006. 10.2105/AJPH.2006.096826 17901444 PMC2377299

[B69] Morris M. W. (2016). *Pushout: The Criminalization of Black Girls in Schools.* New York, NY: The New Press.

[B70] Muthén L. K. Muthén B. O. (1998). *Mplus User’s Guide*, 7th Edn Los Angeles, CA: Muthén & Muthén.

[B71] National Center for Education Statistics (NCES) (2019). *U**.S. Department of Education.* Available online at: https://nces.ed.gov/ (accessed November 15, 2019).

[B72] Navarrete C. D. McDonald M. M. Molina L. E. Sidanius J. (2010). Prejudice at the nexus of race and gender: an outgroup male target hypothesis. *J. Pers. Social Psychol.* 98 933–945. 10.1037/a0017931 20515248

[B73] Nylund K. L. Asparouhov T. Muthén B. O. (2007). Deciding on the number of classes in latent class analysis and growth mixture modeling: a Monte Carlo simulation study. *Struct. Equ. Model. Multidiscip. J.* 14 535–569. 10.1080/10705510701575396

[B74] Ogden C. L. Carroll M. D. Lawman H. G. Fryar C. D. Kruszon-Moran D. Kit B. K. (2016). Trends in obesity prevalence among children and adolescents in the United States, 1988–1994 through 2013-2014. *JAMA* 315 2292–2299. 10.1001/jama.2016.6361 27272581 PMC6361521

[B75] Pettigrew T. F. Tropp L. R. (2006). A meta-analytic test of intergroup contact theory. *J. Pers. Social Psychol.* 90 751–783. 10.1037/0022-3514.90.5.751 16737372

[B76] Pew Research Center (2018). *Teens, Social Media and Technology.* Available online at: https://www.pewresearch.org/internet/2018/05/31/teens-social-media-technology-2018/ (accessed September 25, 2020).

[B77] Phinney J. S. (1992). The multigroup ethnic identity measure a new scale for use with diverse groups. *J. Adolescent Res.* 7 156–176. 10.1177/074355489272003

[B78] Puhl R. Latner J. (2007). Stigma, obesity, and the health of the nation’s children. *Psychol. Bull.* 133 557–580. 10.1037/0033-2909.133.4.557 17592956

[B79] Reinecke J. (2006). Longitudinal analysis of adolescents’ deviant and delinquent behavior. *Methodology* 2 100–112. 10.1027/1614-2241.2.3.100

[B80] Rivas-Drake D. Hughes D. Way N. (2009). A preliminary analysis of associations among ethnic–racial socialization, ethnic discrimination, and ethnic identity among urban sixth graders. *J. Res. Adolescence* 19 558–584. 10.1111/j.1532-7795.2009.00607.x

[B81] Rogers L. O. Meltzoff A. N. (2017). Is gender more important and meaningful than race? An analysis of racial and gender identity among Black, White, and mixed-race children. *Cult. Diversity Ethnic Minority Psychol.* 23 323–334. 10.1037/cdp0000125 27736104

[B82] Ruble D. N. Alvarez J. Bachman M. Cameron J. (2004). “The development of a sense of “we”: the emergence and implications of children’s collective identity,” in *The Development of the Social Self*, eds Bennett M. Sani F. (East Sussex: Psychology Press), 43–90.

[B83] Santos C. E. VanDaalen R. A. (2016). The associations of sexual and ethnic–racial identity commitment, conflicts in allegiances, and mental health among lesbian, gay, and bisexual racial and ethnic minority adults. *J. Counseling Psychol.* 63 668–676. 10.1037/cou0000170 27841452

[B84] Schvey N. Puhl R. Brownell K. (2014). The stress of stigma: exploring the effect of weight stigma on cortisol reactivity. *Psycho. Med.* 76 156–162. 10.1097/PSY.0000000000000031 24434951

[B85] Seaton E. Gee G. Neblett E. Spanierman L. (2018). New directions for racial discrimination research inspired by the integrative model. *Am. Psychol.* 73 768–780. 30188165 10.1037/amp0000315

[B86] Shields S. A. (2008). Gender: an intersectionality perspective. *Sex Roles* 59 301–311. 10.1007/s11199-008-9501-8

[B87] Sidanius J. Pratto F. (eds.) (1999). *Social Dominance: An Intergroup Theory of Social Hierarchy and Oppression.* Cambridge: Cambridge University Press.

[B88] Skiba R. J. Arredondo M. I. Gray C. Rausch M. K. (2016). “What do we know about discipline disparities? New and emerging research,” in *Inequality in School Discipline: Research and Practice to Reduce Disparities*, eds Skiba R. Mediratta K. Rausch K. M. (London: Palgrave MacMillan), 21–38.

[B89] Sladek M. R. Umaña-Taylor A. J. Rivas-Drake D. McDermott E. R. (2020). Testing invariance of ethnic-racial discrimination and identity measures across ethnic-racial groups and contexts. *Psychol. Assess.* 32 509–526. 10.1037/pas0000805 32091231

[B90] Smith D. Schacter H. Enders C. Juvonen J. (2018). Gender norm salience across middle schools: contextual variations in associations between gender atypicality and socioemotional distress. *J. Youth Adolescence* 47 947–960. 10.1007/s10964-017-0732-2 28836082 PMC6128704

[B91] Smith T. Leaper C. (2005). Self-perceived gender typicality and the peer context during adolescence. *J. Res. Adolescence* 16 91–103. 10.1111/j.1532-7795.2006.00123.x

[B92] Smith-Bynum M. A. Lambert S. F. English D. Ialongo N. S. (2014). Associations between trajectories of perceived racial discrimination and psychological symptoms among African American adolescemts. *Dev. Psychopathol.* 26 1049–1065. 10.1017/S0954579414000571 24955844 PMC4205197

[B93] Syed M. Santos C. Yoo H. C. Juang L. P. (2018). Invisibility of racial/ethnic minorities in developmental science: implications for research and institutional practices. *Am. Psychol.* 73 812–826. 10.1037/amp0000294 30188168

[B94] Tajfel H. Turner J. C. (1986). “The social identity theory of intergroup behavior,” in *Psychology of Intergroup Relations*, eds Worchel S. Austin W. (Chicago, IL: Nelson Hall), 7–24.

[B95] Toomey R. B. Ryan C. Diaz R. M. Card N. A. Russell S. T. (2013). Gender-nonconforming lesbian, gay, bisexual, and transgender youth: school victimization and young adult psychosocial adjustment. *Psychol. Sexual Orientation Gender Diversity* 1 71–80. 10.1037/2329-0382.1.S.71 20822214

[B96] Turner K. L. Brown C. S. (2007). The centrality of gender and ethnic identities across individuals and contexts. *Social Dev.* 16 700–719. 10.1111/j.1467-9507.2007.00403.x

[B97] U.S. Census Bureau (2018). *Demographic Turning Points for the United States: Population Projections for 2020 to 2060.* Available online at: https://www.census.gov/content/dam/Census/library/publications/2020/demo/p25-1144.pdf (accessed September 1, 2019).

[B98] Umaña-Taylor A. J. Yazedjian A. Bámaca-Gómez M. (2004). Developing the ethnic identity scale using Eriksonian and social identity perspectives. *Identity Int. J. Theory Res.* 4 9–38.

[B99] Verkuyten M. Thijs J. (2001). Ethnic and gender bias among Dutch and Turkish children in late childhood: the role of social context. *Infant Child Dev.* 10 203–217. 10.1002/icd.279

[B100] Vermunt J. K. (2010). Latent class modeling with covariates: two improved three-step approaches. *Political Anal.* 18 450–469. 10.1093/pan/mpq025

[B101] Way N. Reddy R. Rhodes J. (2007). Students’ perceptions of school climate during the middle school years: associations with trajectories of psychological and behavioral adjustment. *Am. J. Commuity Psychol.* 40 194–213. 10.1007/s10464-007-9143-y 17968655

[B102] Way N. Santos C. Niwa E. Y. Kim-Gervey C. (2008). “To be or not to be: an exploration of ethnic identity development in context,” in *The Intersections of Personal and Social Identities. New Directions for Child and Adolescent Development*, Vol. 120 eds Azmita M. Syed M. Radmacher K. (Hoboken, NJ: Wiley), 61–79. 10.1002/cd.216 18521862

[B103] West B. T. Welch K. B. Galecki A. T. (2006). *Linear Mixed Models – A Practical Guide Using Statistical Software.* London: Chapman & Hall/CRC.

[B104] Yang C. C. Brown B. B. (2013). Motives for using Facebook, patterns of Facebook activities, and late adolescents’ social adjustment to college. *J. Youth Adolescence* 42 403–416. 10.1007/s10964-012-9836-x 23076768

[B105] Yun H. Graham S. (2019). Too tough at the top: using latent class growth analysis to assess cool status during middle school. *J. Adolescence* 75 47–52. 10.1016/j.adolescence.2019.07.001 31326534 PMC6699880

